# Epigenome-wide analysis in newborn blood spots from monozygotic twins discordant for cerebral palsy reveals consistent regional differences in DNA methylation

**DOI:** 10.1186/s13148-018-0457-4

**Published:** 2018-02-23

**Authors:** Namitha Mohandas, Sebastian Bass-Stringer, Jovana Maksimovic, Kylie Crompton, Yuk J. Loke, Janet Walstab, Susan M. Reid, David J. Amor, Dinah Reddihough, Jeffrey M. Craig

**Affiliations:** 1 Environmental and Genetic Epidemiology Research, Murdoch Children’s Research Institute, Royal Children’s Hospital, Flemington Road, Parkville, Victoria 3052 Australia; 2 Department of Paediatrics, The University of Melbourne, Flemington Road, Parkville, Victoria 3052 Australia; 3 Bioinformatics Group, Murdoch Children’s Research Institute, Royal Children’s Hospital, Flemington Road, Parkville, Victoria 3052 Australia; 4 Developmental Disability and Rehabilitation Research, Murdoch Children’s Research Institute, Flemington Road, Parkville, Victoria 3052 Australia; 5 Neurodevelopment and Disability, The Royal Children’s Hospital, Flemington Road, Parkville, Victoria 3052 Australia; 6 Centre for Molecular and Medical Research, School of Medicine, Deakin University, Geelong, Victoria 3220 Australia

**Keywords:** Cerebral palsy, DNA methylation, Inflammation, Epigenetics, Discordant twins

## Abstract

**Background:**

Cerebral palsy (CP) is a clinical description for a group of motor disorders that are heterogeneous with respect to causes, symptoms and severity. A diagnosis of CP cannot usually be made at birth and in some cases may be delayed until 2–3 years of age. This limits opportunities for early intervention that could otherwise improve long-term outcomes. CP has been recorded in monozygotic twins discordant for the disorder, indicating a potential role of non-genetic factors such as intrauterine infection, hypoxia-ischaemia, haemorrhage and thrombosis. The aim of this exploratory study was to utilise the discordant monozygotic twin model to understand and measure epigenetic changes associated with the development of CP.

**Methods:**

We performed a genome-wide analysis of DNA methylation using the Illumina Infinium Human Methylation 450 BeadChip array with DNA from newborn blood spots of 15 monozygotic twin pairs who later became discordant for CP. Quality control and data preprocessing were undertaken using the *minfi* R package. Differential methylation analysis was performed using the remove unwanted variation (RUVm) method, taking twin pairing into account in order to identify CP-specific differentially methylated probes (DMPs), and *bumphunter* was performed to identify differentially methylated regions (DMRs).

**Results:**

We identified 33 top-ranked DMPs based on a nominal *p* value cut-off of *p* < 1 × 10^−4^ and two DMRs (*p* < 1 × 10^−3^) associated with CP. The top-ranked probes related to 25 genes including *HNRNPL*, *RASSF5*, *CD3D* and *KALRN* involved in immune signalling pathways, in addition to *TBC1D24*, *FBXO9* and *VIPR2* previously linked to epileptic encephalopathy. Gene ontology and pathway analysis of top-ranked DMP-associated genes revealed enrichment of inflammatory signalling pathways, regulation of cytokine secretion and regulation of leukocyte-mediated immunity. We also identified two top-ranked DMRs including one on chromosome 6 within the promoter region of *LTA* gene encoding tumour necrosis factor-beta (TNF-β), an important regulator of inflammation and brain development. The second was within the transcription start site of the *LIME1* gene, which plays a key role in inflammatory pathways such as MAPK signalling. CP-specific differential DNA methylation within one of our two top DMRs was validated using an independent platform, MassArray EpiTyper.

**Conclusions:**

Ours is the first epigenome-wide association study of CP in disease-discordant monozygotic twin pairs and suggests a potential role for immune dysfunction in this condition.

**Electronic supplementary material:**

The online version of this article (10.1186/s13148-018-0457-4) contains supplementary material, which is available to authorized users.

## Background

Cerebral palsy (CP) describes a group of motor impairment syndromes caused by lesions or anomalies of the developing brain [1]. It is non-progressive, but the severity of symptoms may change over time [2]. CP is the most common childhood physical disability [3] with a worldwide prevalence of 2.11 per 1000 live births [4]. In preterm infants (< 37 weeks gestation), the prevalence is higher, ranging from 5 to 92 per 1000 depending on gestational age [5]. The prevalence of CP in multiple births is almost four times that of singletons [6]. There are many factors that may be responsible for this increased risk in multiple birth pregnancies, with the most likely being low birth weight and preterm birth, both known risk factors for CP.

The brain insult or anomaly resulting in CP may occur during the prenatal, perinatal or early postnatal period [1, 7], and in many cases, the timing is unknown. Although newborns may be recognised as being at risk of CP, less than half of all children who are ultimately diagnosed are identified before 1 year of age, and only three quarters are identified before age 2 [8].

CP has a multifactorial pathogenesis and risk factors including intrauterine growth restriction or infection, placental abnormalities, inflammation, signs of fetal distress and genetic variation [9, 10]. Although the latter explains a proportion of CP cases, particularly cerebral maldevelopments [9, 11, 12], many non-genetic factors likely play a role [7] though their respective contributions have not been comprehensively addressed [13–15].

Genetically identical monozygotic (MZ) twin pairs discordant for CP highlight the role of non-shared factors in the pathogenesis of CP [16]. Non-shared factors are often described as the differences in the intrauterine environment that influences the development of individual members of a twin pair [17, 18]. Such within-pair variation can arise from differences in the length and morphology of the umbilical cord or placenta. This can affect growth rate and development of the individual twins leading to discordance in infection or inflammation, leading to disease discordance [13, 19, 20]. More generally, the study of phenotypically discordant MZ twins, matched for genetic variation, sex, gestational age and maternal factors, provides a great opportunity to examine the role of epigenetics in disease aetiology by allowing us to isolate the effect of such non-shared environmental factors [21, 22].

Epigenetics refers to a range of modifications and processes that regulate the activity of DNA, including gene expression. Epigenetic variation has emerged as a candidate mediator of a range of health outcomes beginning in early life as part of the ‘Developmental Origins of Health and Disease’ (DOHaD) phenomenon. [23–25]. In DOHaD, environmental conditions in utero and during infancy alter the developmental trajectory of an individual, which manifests as specific chronic health phenotypes later in life.

DNA methylation is the most widely studied epigenetic process and is one of the mechanisms that are involved in tissue differentiation during early development. Despite this, previous studies have investigated the concordance in DNA methylation state between the brain and peripheral tissues, revealing many similarities [26–28]. Several epigenome-wide association studies (EWAS) have identified DNA methylation variation in the cord blood in association with later neurocognitive function and behaviour [29–31]. Similarly, the whole blood has been used to detect differential methylation patterns between affected and unaffected individuals in brain disorders such as schizophrenia [27], bipolar disorder [32, 33] and Alzheimer’s disease [34]. Animal studies have also reported that environmental factors affecting brain processes leave biomarker signatures in the blood with consistent methylation status across the brain and peripheral tissues [35]. We have previously shown that DNA methylation varies within pairs of MZ twins from birth [36, 37]. In this study, we hypothesised that early life non-shared factors that play a role in the aetiology of CP in discordant MZ twins may be reflected in differences in DNA methylation across tissues including neonatal blood. Furthermore, we hypothesised that a subset of differential methylation will be located in genes previously implicated in CP aetiology [38–42], in particular, pathways involved in inflammation, hypoxia-ischaemia and thrombosis.

## Methods

### Samples and DNA extraction

Participant CP-discordant twin pairs, who were suspected to be MZ on the basis of same sex and questionnaire data that measured concordance for hair and eye colour, were recruited through the Victorian Cerebral Palsy Register, a population-based registry of individuals born or receiving medical services in the Australian state of Victoria. Participants were excluded if either twin presented with another known neurological disorder. Written informed consent from the families was obtained. A single 1-cm-diameter dried blood spot was acquired from all participants from a neonatal newborn screening card collected 2 to 4 days after birth and then stored within the Victorian Clinical Genetics Service.

Genomic DNA (gDNA) was extracted from the dried blood spot samples using the ZR DNA Card Extraction Kit (Zymo Research, Irvine, CA, USA) with some modifications to the manufacturer’s protocol. Briefly, eight 3-mm punches were from each 1-cm-diameter blood spot and were transferred to a 2-mL Eppendorf Safe-Lock microcentrifuge tube (Merck, Darmstadt, Germany) containing ZR BashingBeads. Four hundred microliters of PBS containing 40 μL of 20 mg/mL proteinase K (Sigma-Aldrich, St. Louis, Missouri, USA) was added, and samples were vortexed and centrifuged briefly, followed by incubation overnight at 37 °C. Following incubation, 400 μL of ZR lysis solution was added to each tube. Punches were homogenised for 30 s at 4 m/s^2^ using Thermo Savant FastPrep 120 Cell Disrupter System (Global Medical Instrumentation (GMI) Incorporation, Minnesota, USA). Tubes were centrifuged for 1 min at 10,000 rpm, and 390 μL of 2× digestion buffer and 10 μL of 20 mg/mL proteinase K were added. Tubes were mixed by inversion and incubated for 30 min at 55 °C, then left to cool at ambient temperature for 3–4 min before centrifuging for 1 min at 8000 rpm. Six hundred fifty microliters of supernatant was added to 1.3 mL of DNA isolation buffer contained in a 5-mL Falcon tube (Thermo Fisher Scientific, MA, USA). This mixture was passed through the Zymo-Spin IC column by centrifuging for 1 min at 14,000 rpm, followed by the discard of flow-through liquid. The spin column was then washed twice by adding 200 μL of DNA wash buffer and centrifuged for 1 min at 14,000 rpm. Finally, 20 μL of DNA elution buffer (pre-warmed at 55 °C) was added to the column and incubated at ambient temperature for 15 min before final centrifugation for 2 min at 14,000 rpm. This was repeated, resulting in a final elution volume of 40 μL containing genomic DNA. DNA concentration was measured by spectrophotometry (Nanodrop, Wilmington, DE, USA) to allow calculation of the required volume of each sample for array analysis. The quality of the extracted gDNA samples was visualised using agarose gel electrophoresis.

### Illumina Infinium HumanMethylation450 arrays

Following bisulphite conversion of genomic DNA, genome-wide analysis of DNA methylation was assessed using HM450 (Illumina, San Diego, CA, USA), at the Department of Pathology, University of Melbourne. Hybridisation and scanning were performed following the manufacturer’s instructions. Statistical analysis was performed using the R statistical programming language (http://www.R-project.org) in conjunction with Bioconductor packages developed for the analysis of methylation arrays.

### Preprocessing of Illumina Infinium 450K array data

The raw intensity data (IDAT files) were imported into R (3.3.1; http://cran.r-project.org/). Data quality was assessed using the *minfi* (v1.20.2) Bioconductor package [43]. From 485,512 HM450 probes, 67,120 were removed based on either (1) poor performance (mean detection *p* value of > 0.01, *n* = 14,056); (2) probes containing either a single nucleotide polymorphism (SNP) at the target CpG site or at the single nucleotide extension site (*n* = 16,307); (3) probes that map to multiple locations in the genome (*n* = 27,120), [44]; and (4) or to sex chromosomes (*n* = 9637). Samples were also evaluated using a modified version of the Houseman method [45, 46] implemented in *minfi*, to estimate the cell type composition. The Wilcoxon signed-rank statistical test was used to compare the difference in cell type proportion between CP cases and controls. The analysis was completed before a cord blood reference panel was widely available, so cohorts used an adult whole blood reference [43] to estimate the proportion of B cells, CD8+ T-cells, CD4+ T-cells, granulocytes, NK cells and monocytes in each sample. The data was normalised using subset-quantile within array normalisation (SWAN) [47]. Covariates such as birth weight, birth order and postnatal age (in days) at which newborn screening cards were created were assessed as potential confounders.

### Differential methylation analysis

Beta (*β*) values (proportion of the methylated signal over the total signal) were converted to *M*-values, the log_2_ ratio of the intensities of the methylated signal versus the unmethylated signal. Differential methylation analysis was performed using remove unwanted variation (RUVm) [48], implemented in the *missMethyl* R package [49] taking into account the twin relationships. RUVm is a data-driven method of controlling for unwanted technical and biological variation in regression analyses using an empirically determined set of negative control probes assumed not to be associated with the biological factor of interest. *p* values were adjusted to control for the false discovery rate (FDR) using the Benjamini-Hochberg method [50]. Differentially methylated probes (DMPs) were considered significant if they fell within the FDR threshold of 0.1. We also investigated the top-ranked DMPs with an unadjusted *p* value less than 1 × 10^−4^ [51].

### Identification of differentially methylated regions

Differentially methylated regions (DMRs) were identified using the *bumphunter* package [43, 52]. The cut-off value, which is a user-defined numeric value that determines the upper and lower bounds of the genomic profiles that will be used as candidate regions, was set to 0.02 and the number of permutations set to 1000.

### Functional annotation and pathway analysis

Gene ontology and pathway analysis were performed using the *gometh* function from the *missMethyl* package [49], which appropriately takes into account the variable number of HM450 probes associated with each gene. Gene ontology enrichment was performed for the 1000 top-ranked DMP-associated genes. The KEGG option of the *gometh* function in *missMethyl* was used to provide further insights into relevant biological processes associated with the top-ranked DMPs.

### Validation of differentially methylated regions

Site-specific validation was performed using the Sequenom MassArray EpiTYPER (Agena Biosciences). T7-tagged primers were designed for two regions (Additional file 1) using the Sequenom EpiDesigner package [53]. Forward primer sequences contained a 10 base 5′ tag (AGGAAGAGAG) and reverse primers a 31 base 5′ tag (CAGTAATACGACTCACTATAGGGAGAAGGCT). In silico, assay prediction was performed using the BiocLite *MassArray* package. DNA used for validation was the same as that used for the HM450 analysis. Bisulphite treatment of genomic DNA was accomplished using the MethylEasy *Xceed* Kit (Human Genetic Signatures, North Ryde, Australia). One microliter of bisulphite-converted product was amplified in triplicate for each sample using the FastStart kit (Roche, Mannheim, Germany) in 15 μL of reagents with thermal cycling conditions as follows: 95 °C for 10 min; 5 cycles of 95 °C for 10 s, 60 for 30 s and 72 °C for 2 min; 40 cycles of 95 °C for 10 s, 62 for 30 s and 72 °C for 90 s; and final extension at 72 °C for 7 min. Raw data generated from the MassArray EpiTYPER was cleaned using a Microsoft Excel macro developed in-house [54, 55]. The median value of triplicates was determined, and any replicates > 10% from the median were removed as previously described [54, 55].

### Within-twin pair analysis

To explore the top-ranked CpGs within each twin pair and compare them across twin pair groups, probes were ranked according to delta beta (Δ*β*, the difference in DNA methylation of the CP minus non-CP twin) within pairs, and the top-ranked 100 probes were compared across all 15 twin pairs. The genes corresponding to all probes with a Δ*β* value > 0.5 were then compared across the 15 twin pairs. Gene ontology analysis was performed on the top 1000 probes from each twin pair, and common ontologies between twin pairs were identified.

## Results

### Subject characteristics

The study cohort consisted of 16 CP-discordant twin pairs (ten male and six female) for which pre-screening suggested a high probability of monozygosity (Table 1). All were tested for genetic zygosity using data from 65 SNPs from the Infinium arrays. The variability of SNPs for one twin pair (pair no. 9003) was substantially larger than the remaining samples and was therefore assigned as dizygotic (DZ). This pair was excluded from further analysis. Five subtypes of CP were reported: spastic diplegia (6), spastic quadriplegia (3), spastic hemiplegia (3), dyskinesia (2) and ataxia (1). The severity of CP ranged from mild (independently ambulant) to severe (wheelchair dependent), and the underlying neuropathology included white matter (11), grey matter brain injury (2) and both white and grey matter mixed injury (2). Three twin pairs were born at term (37–41 weeks), while all other twin pairs were born preterm (< 37 weeks).

**Table 1:** Descriptive characteristics of the study cohort

Twin ID	Type of CP	Gestational age (weeks)	Brain Injury
9001	Spastic diplegia	27 (Preterm)	WMI
9002	Spastic diplegia	28 (Preterm)	WMI
9003*	Spastic diplegia	29 (Preterm)	WMI
9004	Spastic diplegia	29 (Preterm)	WMI
9005	Spastic quadriplegia	31 (Preterm)	WMI
9006	Spastic diplegia	32 (Preterm)	WMI
9007	Spastic hemiplegia	32 (Preterm)	WMI
9008	Spastic quadriplegia	33 (Preterm)	WMI
9009	Spastic diplegia	33 (Preterm)	WMI
9010	Spastic diplegia	36 (Preterm)	M
9011	Dyskinetic: dystonic	38 (Term)	GMI
9012	Dyskinetic: hypotonia	32 (Preterm)	WMI
9013	Spastic quadriplegia	38 (Term)	M
9014	Ataxia	34 (Preterm)	GMI
9015	Spastic hemiplegia	32 (Preterm)	WMI
9016	Spastic hemiplegia	38 (Term)	WMI

*WMI* white matter injury, *M* miscellaneous, *GMI* grey matter injury

*Twin pair 9003 was later confirmed not to be MZ and removed from the analysis

### Global DNA methylation profiles in CP-discordant monozygotic twins

Global DNA methylation (average *β* value across all probes) within twin pairs was compared using a pairwise Pearson correlation for the 418,392 probes remaining after filtering and quality control (see the ‘Methods’ section) for all 15 twin pairs. Within-pair methylation correlation coefficients ranged from 0.980 to 0.996 (Additional file 2) compared to 0.976 to 0.995 between unrelated unaffected individuals.

### Top-ranked CP-associated DMPs

Cleaned data was explored by principal component (PC) analysis which revealed few (6/54) significant correlations (*p* < 0.05, *r* < 0.6 shaded in Additional file 3) between the top six principal components of DNA methylation and nine technical (e.g. age at which Guthrie card was created) and biological (e.g. sex, subtype of CP) covariates. This suggested that none of the covariates tested was consistently associated with DNA methylation. In addition, none were associated with the largest principal component of variation within the dataset, PC1. Multi dimensional scaling (MDS) plots of the first three dimensions of the processed methylation data also showed that chip location and position on the 450K array were not found to affect methylation data (Additional file 4).

Apart from the above covariates, it is known that cell-type heterogeneity within the whole blood can confound epigenome-wide analyses. Therefore cell-type composition within CP-discordant pairs was evaluated. The levels of CD8^+^ T cells (CD8T) and CD4^+^ T cells (CD4T), B cells, natural-killer (NK) cells, monocytes and granulocytes were compared between the two groups (CP cases and normal co-twins). There was no statistically significant difference in the estimated cell-type proportions of CD8^+^ T cells, NK cells, B cells and monocytes (*p* > 0.05). However, the proportion of CD4^+^ T cells was lower (*p* = 0.002), and the proportion of granulocytes was found higher (*p* = 0.021) in CP cases relative to normal co-twins (Additional file 5).

**Fig. 1:**
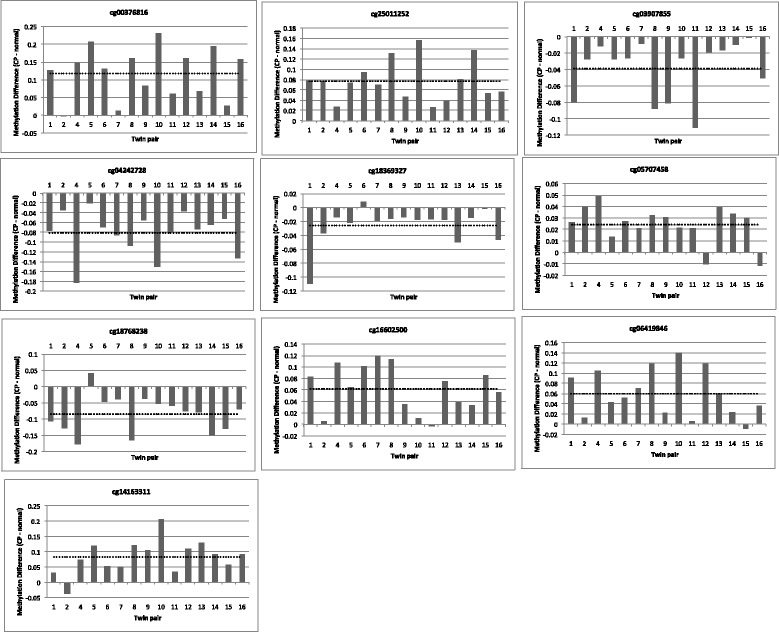
DNA methylation differences (CP minus normal twin) for the top ten CP-specific DMPs. The direction of methylation difference for each probe was consistently in the same direction (hyper- or hypomethylated) across > 12/15 twin pairs. The dotted line represents the average methylation difference, and methylation difference is represented in absolute value (effect size of 0.1 is 10% methylation difference)

To take into account potential sources of unwanted variation (such as cell-type composition), the genome-wide analysis was performed using RUVm, which adjusts for biological and technical variation using a set of data-driven negative control probes [46, 48, 56, 57]. This analysis did not identify any significant CP-associated DMPs after adjusting for multiple testing. Nevertheless, as this is an exploratory epigenome-wide study of CP, we focused on the characteristics of the top-ranked DMPs based on a nominal *p*-value cut-off of *p* < 1 × 10^−4^ as used by others [51]. This resulted in a list of 33 top-ranked DMPs, corresponding to 25 genes (Table 2), most of which showed a consistent direction in the majority of twin pairs (> 12/15). The average difference in methylation (Δ*β* = CP twin minus unaffected twin) ranged from + 0.6 to + 11.9% and from − 2.5 to − 12.4% (Table 2). Figure 1 shows the within-pair differences in methylation for the top ten DMPs.

**Table 2:** The top-ranked DMPs (ranked on unadjusted *p* value) in cerebral palsy discordant twins

Probe ID	Rank	Chromosome position	Gene	Location	*P* value	Methylation difference (%)
cg00376816	1	chr19: 39332571	*HNRNPL*		4.57E-06	11.64
cg25011252	2	chr8: 61777859	*CHD7*		6.33E-06	7.64
cg04242728	3	chr16: 2536153	*TBC1D24*		1.46E-05	− 8.20
cg03907855	4	chr8: 1106363	–	Island	1.52E-05	− 3.20
cg05707458	5	chr1: 205424829	–	Island	1.93E-05	2.39
cg18369327	6	chr5: 109220986	*LOC100289673*		2.07E-05	− 2.54
cg18768238	7	chr10: 49892829	*WDFY4*		2.52E-05	− 8.58
cg14163311	8	chr1: 206730397	*RASSF5*		3.21E-05	8.20
cg16602500	9	chr1: 52889575	*ZCCHC11*		3.64E-05	6.25
cg06419846	10	chr11: 66083572	*CD248*		3.65E-05	5.95
cg03929569	11	chr13: 30688864	–	Island	4.35E-05	0.60
cg14414943	12	chr1: 111770718	*CHI3L2*		4.65E-05	6.21
cg19607845	13	chr6: 52930050	*FBXO9*		4.79E-05	− 9.61
cg19942731	14	chr22: 45609421	*C22orf9*		5.11E-05	− 10.38
cg14073571	15	chr7: 158823178	*VIPR2*		5.14E-05	− 7.59
cg03106245	16	chr11: 47399788	*SPI1*		5.33E-05	− 11.45
cg08936645	17	chr4: 37910273	*TBC1D1*		5.36E-05	− 7.84
cg02806715	18	chr6: 32920567	*HLA-DMA*		5.69E-05	− 6.58
cg17512380	19	chr10: 18971598	–	OpenSea	5.81E-05	5.67
ch.10.89216809R	20	chr10: 89226829	–	OpenSea	6.52E-05	− 3.85
cg15613292	21	chr2: 232478359	–	Shore	6.89E-05	2.62
cg00263248	22	chr14: 98444151	*C14orf64*		6.94E-05	6.40
cg19540797	23	chr2: 3605489	*RNASEH1*		7.30E-05	2.27
cg08360638	24	chr13: 20781097	–	OpenSea	7.52E-05	7.09
cg07728874	25	chr11: 118213272	*CD3D*		7.93E-05	9.14
cg11348257	26	chr1: 76556226	*ST6GALNAC3*		8.03E-05	− 12.35
cg07011093	27	chr3: 123987726	*KALRN*		8.38E-05	− 5.03
cg09335613	28	chr6: 71998106	*OGFRL1*		8.85E-05	− 6.68
cg22230912	29	chr3: 16331335	*OXNAD1*		8.90E-05	11.86
cg13505608	30	chr9: 140128562	*SLC34A3*		9.22E-05	2.53
cg04975778	31	chr2: 62732758	*TMEM17*		9.26E-05	2.31
cg12306086	32	chr4: 106117747	*TET2*		9.52E-05	− 10.22

Methylation difference (%) was calculated as the mean of the DNA methylation levels of the CP twin minus the unaffected twin (Δ*β*)

The top-ranked probe *cg00376816* (average Δ*β* = 11.6%, *p* = 4.57 × 10^−6^) was located on chromosome 19, within the gene body of the *HNRNPL* gene encoding the heterogeneous nuclear ribonucleoprotein L. Others included *cg04242728* (in the 5′ end of the *TBC1D24* gene, ranked 3) and *cg19607845* (in the gene body of *FBXO9* gene, ranked 13). Probes located near the gene body of immune and inflammatory genes, such as Ras association domain family member 5 (*RASSF5*), major histocompatibility complex DM alpha-chain (*HLA-DMA*), *CD3D* and kalirin (*KALRN*) genes, were also among the top-ranked 33 DMPs.

To identify enriched biological processes or molecular functions, we performed gene ontology analysis on genes associated with the top-ranked 1000 DMPs. The top 20 gene ontologies ranked by nominal *p*-value were ‘regulation of immune response’, ‘lymphocyte activation’, ‘differentiation and aggregation and T cell activation’ with the top two ontologies related to cell-cell adhesion processes (Table 3; Additional file 6). Enriched disease pathways, as reported by KEGG analysis, included MAPK signalling (19 associated genes from 245 genes in the KEGG pathway list; *p* value 3.6 × 10^−10^), cytokine-cytokine receptor interaction (13 associated genes from 240 genes; *p* value 1.3 × 10^−08^) and Ras signalling (15 associated genes from 218 genes; *p* value: 9.8 × 10^−08^).

**Table 3:** Top 20 gene ontology (GO) terms (BP = biological process) analysed for the 1000 top-ranked CP-associated DMPs

GO ID	GO term	Ontology	No. of genes	DM genes	Unadjusted *p* value
GO:0098609	Cell-cell adhesion	BP	1091	90	8.27E-06
GO:0007156	Homophilic cell adhesion via plasma membrane adhesion molecules	BP	149	25	1.70E-05
GO:0042098	T cell proliferation	BP	157	18	2.91E-05
GO:0042129	Regulation of T cell proliferation	BP	133	16	3.28E-05
GO:0046649	Lymphocyte activation	BP	557	46	6.47E-05
GO:0032729	Positive regulation of interferon-gamma production	BP	58	10	7.55E-05
GO:0070661	Leukocyte proliferation	BP	241	23	9.24E-05
GO:0001775	Cell activation	BP	811	61	9.70E-05
GO:0050863	Regulation of T cell activation	BP	270	26	1.27E-04
GO:0022610	Biological adhesion	BP	1611	117	1.28E-04
GO:1903037	Regulation of leukocyte cell-cell adhesion	BP	284	27	1.30E-04
GO:0070663	Regulation of leukocyte proliferation	BP	187	19	1.66E-04
GO:0045321	Leukocyte activation	BP	656	50	1.93E-04
GO:0007159	Leukocyte cell-cell adhesion	BP	442	37	2.27E-04
GO:0098742	Cell-cell adhesion via plasma-membrane adhesion molecules	BP	214	27	2.32E-04
GO:0050865	Regulation of cell activation	BP	437	37	2.37E-04
GO:0016337	Single organismal cell-cell adhesion	BP	669	54	2.49E-04
GO:0032649	Regulation of interferon-gamma production	BP	85	11	2.51E-04
GO:0007155	Cell adhesion	BP	1606	115	2.56E-04
GO:0045601	Regulation of endothelial cell differentiation	BP	28	7	2.75E-04

*DM* differentially methylated, *FDR* false discovery rate

**Fig. 2:**
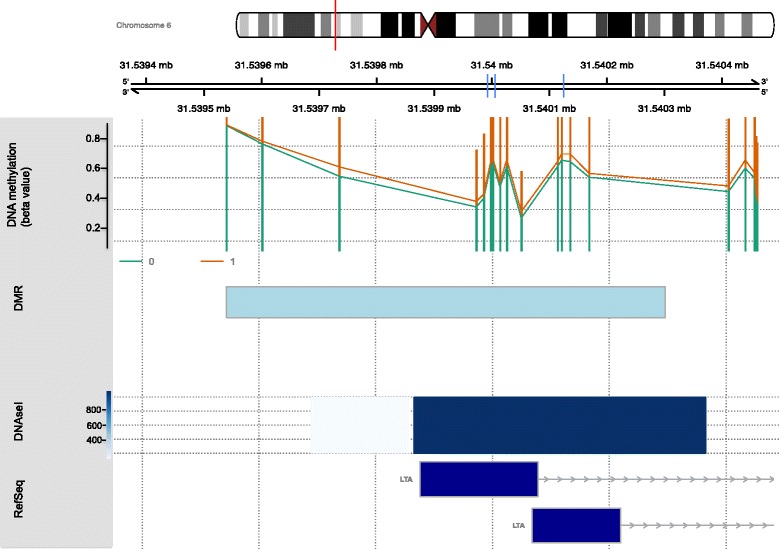
Schematic representation of the top-ranked DMR (*LTA*). The genomic coordinates are plotted on the top lane using the version hg19 of the UCSC genome browser (https://genome.ucsc.edu), where each division is 200 base pairs. Mean beta values for CP (red line) and normal (green lines) twins are plotted on the next lane. The light blue box indicates the differentially methylated region. DNase I hypersensitive sites is plotted in subsequent lane. The DNase I hypersensitive site data was obtained from the UCSC Genome Browser, and the dark blue boxes represent the characterised gene based on reference sequence. The vertical blue lines on the genome browser map indicate the three CpG units validated on MassArray EpiTYPER

We also identified DMRs associated with CP [52] (Table 4). The top-ranked DMR, spanning 434 bp and with a *p* value of 5.6 × 10^−4^, was located on chromosome 6 (Fig. 2). This DMR spans 12 probes (average Δ*β* = 3.7%) within the coding region of the *LTA* gene, approximately ~ 800 bp downstream of the transcription start site (TSS). *LTA* codes for the lymphotoxin-alpha protein otherwise known as tumour necrosis factor beta (TNF-β). Other top DMRs include those within *LTBP1*, *CD300*, *CHST11* and *LIME1*. Gene ontology analysis of the top-ranked DMR-associated genes revealed similar findings to top-ranked DMPs (Table 5; Additional file 7). We found an over-representation of inflammatory signalling pathways, also similar to that of the top-ranked DMPs. The top pathways included TNF and TGF-beta signalling and cytokine-cytokine receptor interaction. The nuclear factor kappa-light-chain-enhancer of activated B cell (NF-κB) signalling pathway was also enriched.

**Table 4:** Differentially methylated regions (DMRs) from Bumphunter analysis

Chromosome position	Gene	Gene description	*P* value	Fwer
chr6: 31539735-31540169	*LTA*	Lymphotoxin-alpha	0.00056	0.988
chr12: 31799116-31799118	–	–	0.00125	0.992
chr11: 47399813-47400146	*SPI1*	Spi-1 Proto-Oncogene	0.00129	0.996
chr2: 33359198-33359688	*LTBP1*	Latent transforming growth factor beta binding protein 1	0.00166	0.996
chr17: 72527607-72527724	*CD300LB*	CD300 molecule like family member b	0.00244	0.996
chr12: 105071483-105071483	*CHST11*	Carbohydrate chondroitin 4 sulfotransferase 11	0.00259	0.996
chr9: 130868874-130868874	*SLC25A25*	Solute carrier family 25 member 25	0.00269	0.996
chr11: 2722062-2722195	*KCNQ1*	Potassium voltage-gated channel subfamily Q member 1	0.00164	1
chr1: 27961563-27962037	*FGR*	FGR proto-oncogene, Src family tyrosine kinase	0.00223	1
chr20: 62367805-62367893	*LIME1*	Lck interacting transmembrane adaptor 1	0.00167	1

*fwer* family-wise error rate

**Table 5:** Gene ontology (GO) analysis for cerebral palsy-associated DMRs. (DM: differentially methylated; FDR: false discovery rate)

GO ID	GO term	Ontology	No. of genes	DM genes	Unadjusted *p* value	FDR
GO:0002705	Positive regulation of leukocyte mediated immunity	BP	80	5	4.57E-10	9.60E-06
GO:0002703	Regulation of leukocyte mediated immunity	BP	139	5	7.48E-09	5.26E-05
GO:0002699	Positive regulation of immune effector process	BP	142	5	7.52E-09	5.26E-05
GO:0050715	Positive regulation of cytokine secretion	BP	91	4	8.69E-08	4.56E-04
GO:0002443	Leukocyte mediated immunity	BP	258	5	1.42E-07	5.95E-04
GO:0002697	Regulation of immune effector process	BP	291	5	4.04E-07	1.09E-03
GO:0042742	Defence response to bacterium	BP	204	4	4.13E-07	1.09E-03
GO:0050778	Positive regulation of immune response	BP	571	6	4.17E-07	1.09E-03
GO:0050707	Regulation of cytokine secretion	BP	135	4	5.96E-07	1.39E-03
GO:0050663	Cytokine secretion	BP	154	4	9.84E-07	2.06E-03
GO:0001819	Positive regulation of cytokine production	BP	360	5	1.32E-06	2.52E-03
GO:0002876	Positive regulation of chronic inflammatory Response to antigenic stimulus	BP	2	2	1.61E-06	2.81E-03
GO:0050776	Regulation of immune response	BP	769	6	1.82E-06	2.82E-03
GO:0002682	Regulation of immune system process	BP	1191	7	1.88E-06	2.82E-03
GO:0002874	Regulation of chronic inflammatory response to antigenic stimulus	BP	3	2	2.15E-06	3.00E-03
GO:0050830	Defence response to Gram-positive bacterium	BP	68	3	2.45E-06	3.22E-03
GO:0002925	Positive regulation of humoral immune response mediated by circulating immunoglobulin	BP	4	2	2.82E-06	3.48E-03
GO:0002678	Positive regulation of chronic inflammatory response	BP	4	2	3.23E-06	3.76E-03
GO:0002718	Regulation of cytokine production involved in immune response	BP	52	3	3.84E-06	4.24E-03
GO:0002684	Positive regulation of immune system process	BP	820	6	4.24E-06	4.44E-03

### Validation of DMRs

*LIME1* and *LTA* DMRs were selected for validation as they were highly ranked, had large, consistent effect sizes across all pairs and were biologically relevant to CP. Three CpGs from the HM450 platform contained within three CpG units on the MassArray Epityper (consisting of seven CpG sites in total) were tested for the *LIME1* DMR, and four CpGs contained within three units on the MassArray Epityper (four CpG sites in total) were tested for the *LTA* DMR, both in regions being approximately 200 base pairs upstream of the transcriptional start site (TSS200) and likely to be in gene promoters. Scatter plots were generated to assess the validity of the data for the three *LIME1* and *LTA* probes within a 250-base-pair region across both the HM450 and the EpiTYPER platforms (Additional file 8). Pearson’s correlation coefficients were determined, and the significance of the correlation was assessed for each probe-CpG unit comparison. Five out of six probes (*cg21201401*, *cg06653796*, *cg14597739*, *cg11586857*, *cg21999229*) had a positive correlation between the two platforms. Among these, one probe out of three for *LIME1* and two of three for *LTA* had moderate correlations (*r* > 0.5). All moderate correlations were also significant with *p* < 0.05 (*r* = 0.88, *p* = 1.6 × 10^−4^; *r* = 0.40, *p* = 0.197; *r* = 0.12, *p* = 0.65 for *LIME1* and *r* = 0.69, *p* = 1.4 × 10^−4^; *r* = 0.59, *p* = 0.043; *r* = 0.21, *p* = 0.34 for *LTA*.). The Δ*β* values were calculated for two of the three probes within the *LTA* gene region, with correlation coefficient values of *r* = 0.69 for *cg14597739* and *r* = 0.21 for *cg21999229*. The Δ*β* for probes within the *LIME1* gene region were not calculated due to insufficient data points, resulting from limited material remaining, for a valid within-pair analysis.

### Differential methylation analysis within individual twin pairs

Since CP is a highly heterogeneous disorder [58], it is possible that a subset of disease-associated DNA methylation patterns may be specific to each proband. To test this [59], we determined the top CP-associated CpGs for each twin pair ranked by absolute differences in DNA methylation (Δ*β* > 0.5) and looked at significant gene ontologies common to multiple pairs (Additional file 9). Gene ontologies corresponding to cell adhesion were found in 5/15 twin pairs (Additional file 10). Similarly, common CpGs with a within-pair methylation difference > 50% were found in multiple twin pairs (Table 6) in genes such as *BICD2*, *HLA-DPB2*, *RPTOR* and *PIK3CG* (Additional file 11), involved in neuronal cell migration, muscular atrophy or muscle contraction pathways and immune response and inflammatory pathways, respectively [60–63]. Notably, two different CpG sites within twin pairs 4 and 8 corresponding to the *WWTR1* gene had an absolute methylation difference of greater than 50% with the same direction of effect. Affected CpG sites were located near the 5′ end of the gene in both pairs (Table 6).

**Table 6:** Genes/probes with an absolute methylation difference of > 50% common to multiple twin pairs

Twin pair group	Methylation difference	Common genes
TW7	0.784	*MED27* (*cg26228569*)
TW15	− 0.749	
TW5	0.824	*BICD2* (*cg14341177*)
TW6	− 0.796	
TW7	0.705	
TW1	0.534	*HLA-DPB2* (*cg01309395*)
TW2	− 0.627	
TW11	0.693	
TW10	0.585	
TW2	− 0.768	*CNOT6L* (*cg11671265*)
TW4	0.745	
TW2	0.634	*SIM1* (*cg00736459*)
TW4	0.556	
TW2	− 0.752	*NR2C2* (*ch.3.343413R*)
TW14	0.584	
TW2	0.753	*LRP11* (*cg24761195*)
TW16	0.811	
TW4	− 0.554	*WWTR1* (*cg02134705*)
TW8	− 0.773	*WWTR1* (*cg19547293*)
TW8	− 0.746	*HYAL3;NAT6* (*cg13682223*)
TW14	0.872	
TW5	− 0.707	
TW9	0.797	*RPTOR* (*cg08905415*)
TW14	− 0.748	
TW4	0.615	*PIK3CG* (*cg08779777*)
TW16	− 0.505	
TW10	− 0.678	

## Discussion

This exploratory study represents an initial step towards investigating potential CP-associated epigenetic differences, with the longer-term aim of identifying predictive biomarkers with clinical utility. We identified DNA methylation differences in dried blood spots from 15 CP-discordant MZ twin pairs and found differential methylation at several gene loci associated with hypoxia signalling, inflammation and cell adhesion. These pathways had been previously linked to CP, consistent with part of our hypothesis.

Pairwise global DNA methylation difference between CP and non-CP members of each pair measured for each twin group and comparison of the size of DNA methylation difference made between groups allowed for an assessment of how variable the differences in methylation may be for different cases of CP. Our results are consistent with previous studies (e.g. [59]) that have indicated that neurodevelopmental disorders such as autism spectrum disorder are not associated with systemic within-twin pair differences in global DNA methylation.

We tested site-specific DNA methylation patterns across the genome for their association with CP, taking CP status within discordant MZ pairs into account. Although no probe reached an adjusted statistical significance at FDR < 0.1, the top-ranked DMPs, at a nominal cut-off of *p* < 1 × 10^−4^, were enriched for the cellular processes of inflammation, cell adhesion and immune response. These showed the direction of effect across most or all discordant twin pairs. This was in accordance with EWAS of other neurodevelopmental disorders including twins discordant for autism spectrum disorders [59], asthma [51], depression [64] and aggressive behaviour [65]. Average within-pair DNA methylation differences of up to 12.5% were observed, comparable to previous findings in neurodevelopmental disorders, ranging from 1.5 to 12%. Furthermore, we validated five of the six CpG probes, with positive correlations across platforms. Three of these five probes (one from *LIME1* and two from *LTA*) showed a strong and significant cross-platform correlation, indicating the validity of methylation values between platforms.

Genes associated with top-ranked DMPs and DMRs were enriched for similar ontologies and pathways, namely immune response, lymphocyte-mediated immunity, interferon-gamma production and regulation of immune response.

Our study revealed top-ranked DMRs associated with genes that play a role in inflammation, such as *LTA/TNF*β and *LIME1*, supporting part of our hypothesis that inflammation plays a key role in CP aetiology. Genetic variants of *LTA* have been implicated in multiple studies as being associated with risk for CP [66–68]. *LTA* plays an important role in inflammation and brain development, mediating preterm birth and white matter brain injury [69]. It has been implicated that inflammation [70] and increased levels of its isoform TNF-α were found in children with CP compared to healthy controls [71]. *LIME1* gene links T and B cell signalling to the activation of tyrosine and MAP kinases [72]. Other top DMR-associated genes such as *LTBP1* and *CD300* are also known mediators of inflammatory pathways such as ERK signalling pathway, interleukin-3,-5 pathway, B cell receptor signalling pathway and other chemokine signalling [72–74]. Genes associated with top-ranked DMPs also showed involvement in other key inflammatory pathways such as Ras signalling (*WDFY4*), MAP kinase signalling (*CD3D*) and interleukin-3,-5 signalling (*KALRN*). Analysis within individual twin pairs also revealed associations to the *WWTR1* gene, which is involved in the activation of TGF-β signalling pathway, an inflammatory pathway that regulates neural survival and death [75, 76]. Gene ontology analysis of top-ranked DMPs showed enrichment of genes involved in regulation of immune response pathways such as those involved in signalling or T-cell activation. It is noteworthy that intrauterine infection is a known risk factor for CP and that many inflammatory cytokines have been shown to be critical to the risks associated with CP [38, 41, 60].

Perinatal brain injury can be induced by a range of insults such as hypoxic-ischaemic injury or infection [77]. An in utero infection such as chorioamnionitis may trigger an innate immune response, resulting in elevated cytokine levels. Cytokines in the fetal blood may contribute to a systemic fetal inflammatory response with eventual penetration across the blood-brain barrier resulting in an inflammatory cascade in the fetal brain [78]. Brain injury induced by neonatal hypoxia-ischaemia also involves key components of inflammation such as immune cells, chemokines, cytokines and cell adhesion molecules [79]. Therefore, we suggest that inflammation may play a role in perinatal brain damage associated with CP.

We also observed an enrichment of CP-associated DMPs and DMRs in gene ontologies associated with cell adhesion, and this was also observed in individual twin pairs. Aberrant expression of cell adhesion molecules has been reported in muscle biopsies of both children and adults with CP [39]. Previous whole exome and whole genome sequencing studies have also illustrated the potential role of cell adhesion in CP by identifying genetic variants in novel candidate genes which function as neural adhesion molecules essential for neurite outgrowth and axon guidance [9, 12]. The NF-κB transcription factor signalling pathway was common in both DMPs and DMRs.

Our results are consistent with a previous gene expression study in newborn blood spot samples from children with CP [80], which identified up-regulation of inflammatory pathways in preterm children who later developed the disorder. Other similarities between the two studies include variation in genes involved in T-cell and B-cell receptor signalling pathways and cytokine-cytokine receptor interaction, all of which were shown to have a dysregulation in CP cases [80]. However, we found no evidence for an association with increased thyroid function in preterm-born CP cases as hypothesised and reported previously [80].

Another top-ranked DMP was *HNRNPL*, which likely plays a role in response to hypoxia via regulation of the vascular endothelial growth factor (*VEGF*) gene [81]. Hypoxia is known to hinder normal development and maturation of the brain and can cause white matter injury in preterm born infants [82] resulting in CP [83]. This finding supports our hypothesis that epigenetic alterations in genes involved in hypoxic pathways play a role in the aetiology of CP.

Two high-ranking DMPs lie within the *TBC1D24* and *FBOX9* genes respectively, and both have previously been associated with epilepsy [84, 85]. In 29% of CP cases in Victoria, Australia, epilepsy is comorbid with CP [5]. These results may suggest a potentially shared aetiology between epilepsy and CP [86, 87].

Our findings agree with those of others showing a link between early life DNA methylation state and neurodevelopmental and cognitive outcomes [29, 88], which would allow for early diagnosis and facilitate timely intervention. Currently, MRI scans, assessment tests such as the General Movements Assessment and interventions such as environmental enrichment, early developmental, early motor and physiotherapy interventions are used to inform strategies for early intervention in high-risk groups, such as preterm born children, [89–93].

Given that CP is a highly heterogeneous condition, this study highlights the importance of using epigenetic biomarkers to distinguish and detect underlying pathways across the disorder. For individuals, such an epigenetic state at birth could be used to estimate risk for subsequent development of overt CP.

The strength of using MZ twins is that they are matched for parental age, age, sex, season of birth and genetic factors. Although twins have a higher risk of CP than singletons, the causative mechanisms, such as thrombosis, and infection in the mother, the placenta or the umbilical cords are likely to be similar [7]. Studying twins discordant for CP allows genetic and environmental components to be partitioned from each other and provides a unique opportunity to evaluate the importance of non-shared environmental factors such as umbilical cord or placental function during early development in isolation. It is possible that only twin of a pair may develop an infection or inflammation of the umbilical cord or placenta ([13, 19, 20, 94]). Such non-shared environmental factors are known to influence the development of individual members of a twin pair. This pilot study also highlights the importance of analysing DNA methylation in dried blood spots, which are collected at birth and stored by many countries and the potential for developing future predictive diagnostic tests [55, 95, 96].

Although each tissue has a subset of CpGs whose DNA methylation patterns are tissue-specific, DNA methylation changes that are concordant between the blood and brain have been detected in previous studies [32, 97, 98]. One example is where they identified parallel changes in DNA methylation between the brain and blood in 30% of the genes implicated in Parkinson’s disease [98]. It was also shown that a DNA methylation module exists in key ageing-related regulatory genes both in the brain and blood [99]. In addition, animal studies have reported that an early environment resulting in a brain disorder can alter DNA methylation in the same gene across the brain and peripheral tissues [35].

There are some limitations to this study. While similar in sample size to many comparable twin studies of brain-related disorders [58, 59, 64, 100], we acknowledge that larger sample sizes of 25 twin pairs or more are preferable to detect a mean effect size of at least 8% methylation (FDR = 0.05) [101]. As CP is a heterogeneous condition, the small sample size of our cohort, with a lack of CP concordant and healthy twin pairs for comparison, also limits our capability to understand the biological mechanisms of brain injury that may be specifically associated with CP subtype. The use of peripheral tissue and blood also limits our capability to pinpoint the mechanism of CP. Despite the fact that the brain and blood arise from separate cell lineages, and are thought to be epigenetically distinct, many epigenetic studies are often conducted in the blood due to ease of availability [102]. Previous investigations of methylomic variation across the blood and brain tissue from different regions of the brain have found distinct differences in gene expression and DNA methylation patterns [26, 103–105]. Studies have also shown the inconsistencies in DNA methylation markers from the blood in predicting brain DNA methylation status [27, 106]. However, evidence from animal studies have also shown that blood DNA methylation patterns may in fact reflect patterns in the brain in a subset of genes [35, 107], suggesting that peripheral epigenetic marks may reflect disease mechanisms in some cases. Examples where methylation levels correlate between blood and brain have been reported in Parkinson’s disease, depression, schizophrenia, bipolar disorder and autism [107, 108]. The blood is also particularly useful in investigating disease biomarkers [98] and is an important peripheral tissue to consider for neurological disorders, as it is easily accessible to assist in diagnosis. Another limitation is that our data apply to twins only, and we cannot yet generalise our findings more broadly, as there is evidence that risk factors and associated mechanisms leading to CP may be different in twins compared to singletons [109]. To overcome this, our analysis will be repeated in further sets of twins and singletons. Only with this information can we then start to put together risk models for predicting CP at the time of birth. This approach will provide a unique opportunity to identify a biomarker to predict neurodevelopmental outcomes such as CP.

## Conclusion

This study provides the first evidence that environment-mediated differential methylation in genes involved in known processes such as hypoxia and inflammation, and perhaps processes such as cell adhesion, may contribute to the development of CP. Our data also pave the way for larger studies to use DNA methylation data in risk models to help predict CP before the onset of overt symptoms and therefore provide a chance for timely ameliorative interventions.

## Additional file

Additional file 1: Primer sequences used in site-specific validation using MassArray EpiTYPER. (XLSX 8 kb)

Additional file 2: Scatter plots of genome-wide DNA methylation discordance within twin groups. (PPTX 331 kb)

Additional file 3: Heat map of the associations between the six largest principal components and specified covariates. The heat map provides a score of the strength of the association between DNA methylation (using *M* values) and each covariate, with positive and negative correlations ranging according to the magnitude (red positive, blue negative). The values in brackets for each association represent the *p*-value of the correlation. Of the six significant (*p* < 0.05) associations, all are weak (correlation < 0.6). Abbreviations: CP, cerebral palsy; PC, principal component; PIC, person in charge of performing DNA extraction; GA, gestational age; GMFCS, gross motor function classification system; Guthrie age, age in postnatal days when Guthrie card was made. (PDF 53 kb)

Additional file 4: MDS plots for preprocessed data. Samples are coloured based on chip location ranging from 1 to 3. The figure represents similarities between samples’ 1000 most variable probes based on Euclidean distance (sum of squared differences). Dimension 1 represents the largest variation in the dataset, and 2 and 3 are the second and third largest, respectively. (PDF 42 kb)

Additional file 5: Comparison of cell type composition of cerebral palsy cases versus normal individuals. CD8T and CD4T: cytotoxic T cells; NK: natural-killer cells; B cell: B cell or B lymphocytes; Mono: monocytes; Gran: granulocytes. (PDF 87 kb)

Additional file 6: Gene ontology (GO) analysis for top-ranked 1000 DMPs ranked by *p* value. (XLSX 20 kb)

Additional file 7: Gene ontology (GO) analysis for top DMRs ranked by p-value (XLSX 38 kb)

Additional file 8: Cross-platform validation of the two top DMRs, *LTA* and *LIME1*, between HM450 and EpiTYPER platforms. Pearson’s correlation coefficients for each probe are shown. The scale of both axes reflects a methylation value between 0 and 1 (*β*). The regression lines are shown in black. Based on the *r* value (correlation coefficient), correlations across both platforms are shown. The *p*-value indicates the significance of the correlation. (ZIP 126 kb)

Additional file 9: DNA methylation differences within each discordant CP twin pair, identifying numerous loci showing large DNA methylation differences within each discordant twin pair. (PPTX 3104 kb)

Additional file 10: Gene ontology (biological process) common to multiple twin pairs. (XLSX 21 kb)

Additional file 11: CpG sites (probes) within each twin pair group with an absolute methylation difference > 0.5 and their corresponding genes. Genes are colour coded to highlight overlaps between twin pair groups. (XLSX 30 kb)

## Abbreviations

CP: Cerebral palsy
DMP: Differentially methylated probe
DMR: Differentially methylated region
EWAS: Epigenome-wide association study
HM450: Human Methylation 450 BeadChip array
MZ: Monozygotic
RUV: Remove unwanted variation
SNP: Single nucleotide polymorphism
SWAN: Subset-quantile within array normalisation

## References

[1] Rosenbaum P, Paneth N, Leviton A, Goldstein M, Bax M (2007). A report: the definition and classification of cerebral palsy April 2006. Dev Med Child Neurol Suppl.

[2] Morris C (2007). Definition and classification of cerebral palsy: a historical perspective. Dev Med Child Neurol Suppl.

[3] Novak I, Hines M, Goldsmith S, Barclay R (2012). Clinical prognostic messages from a systematic review on cerebral palsy. Pediatrics.

[4] Oskoui M, Coutinho F, Dykeman J, Jette N, Pringsheim T (2013). An update on the prevalence of cerebral palsy: a systematic review and meta-analysis. Dev Med Child Neurol.

[5] Reid SM, Meehan E, McIntyre S, Goldsmith S, Badawi N, Reddihough DS, Australian Cerebral Palsy Register G (2016). Temporal trends in cerebral palsy by impairment severity and birth gestation. Dev Med Child Neurol.

[6] Smithers-Sheedy H, McIntyre S, Gibson C, Meehan E, Scott H, Goldsmith S, Watson L, Badawi N, Walker K, Novak I (2016). A special supplement: findings from the Australian Cerebral Palsy Register, birth years 1993 to 2006. Dev Med Child Neurol.

[7] Pharoah PO, Dundar Y (2009). Monozygotic twinning, cerebral palsy and congenital anomalies. Hum Reprod Update.

[8] ACPR group. Report of the Australian Cerebral Palsy Register, birth years 1993-2009. Sydney: Cerebral Palsy Alliance Research Institute; 2016.

[9] McMichael G, Bainbridge MN, Haan E, Corbett M, Gardner A, Thompson S, van Bon BW, van Eyk CL, Broadbent J, Reynolds C (2015). Whole-exome sequencing points to considerable genetic heterogeneity of cerebral palsy. Mol Psychiatry.

[10] Keogh JM, Badawi N (2006). The origins of cerebral palsy. Curr Opin Neurol.

[11] O’Callaghan ME, MacLennan AH, Haan EA, Dekker G, South Australian Cerebral Palsy Research G (2009). The genomic basis of cerebral palsy: a HuGE systematic literature review. Hum Genet.

[12] Oskoui M, Gazzellone MJ, Thiruvahindrapuram B, Zarrei M, Andersen J, Wei J, Wang Z, Wintle RF, Marshall CR, Cohn RD (2015). Clinically relevant copy number variations detected in cerebral palsy. Nat Commun.

[13] Phung DT, Blickstein I, Goldman RD, Machin GA, LoSasso RD, Keith LG (2002). The northwestern twin Chorionicity study I. Discordant inflammatory findings that are related to chorionicity in presenting versus nonpresenting twins. Am J Obstet Gynecol.

[14] Livinec F, Ancel PY, Marret S, Arnaud C, Fresson J, Pierrat V, Roze JC, Escande B, Thiriez G, Larroque B (2005). Prenatal risk factors for cerebral palsy in very preterm singletons and twins. Obstet Gynecol.

[15] Benirschke K (1995). The biology of the twinning process: how placentation influences outcome. Semin Perinatol.

[16] Vinnars MT, Vollmer B, Nasiell J, Papadogiannakis N, Westgren M (2015). Association between cerebral palsy and microscopically verified placental infarction in extremely preterm infants. Acta Obstet Gynecol Scand.

[17] Stromswold K (2006). Why aren’t identical twins linguistically identical? Genetic, prenatal and postnatal factors. Cognition.

[18] Plomin R (2011). Commentary: why are children in the same family so different? Non-shared environment three decades later.

[19] Dickinson JE, Keil AD, Charles AK (2006). Discordant fetal infection for parvovirus B19 in a dichorionic twin pregnancy. Twin Res Hum Genet.

[20] Kamran Yusuf HJK (2008). The fetus, not the mother, elicits maternal immunologic rejection: lessons from discordant dizygotic twin placentas.

[21] Boomsma D, Busjahn A, Peltonen L (2002). Classical twin studies and beyond. Nat Rev Genet.

[22] van Dongen J, Slagboom PE, Draisma HH, Martin NG, Boomsma DI (2012). The continuing value of twin studies in the omics era. Nat Rev Genet.

[23] Barker DJ, Winter PD, Osmond C, Margetts B, Simmonds SJ (1989). Weight in infancy and death from ischaemic heart disease. Lancet.

[24] Barker DJ, Osmond C (1986). Infant mortality, childhood nutrition, and ischaemic heart disease in England and Wales. Lancet.

[25] Barker DJ, Gluckman PD, Godfrey KM, Harding JE, Owens JA, Robinson JS (1993). Fetal nutrition and cardiovascular disease in adult life. Lancet.

[26] Davies MN, Volta M, Pidsley R, Lunnon K, Dixit A, Lovestone S, Coarfa C, Harris RA, Milosavljevic A, Troakes C (2012). Functional annotation of the human brain methylome identifies tissue-specific epigenetic variation across brain and blood. Genome Biol.

[27] Walton E, Hass J, Liu J, Roffman JL, Bernardoni F, Roessner V, Kirsch M, Schackert G, Calhoun V, Ehrlich S (2016). Correspondence of DNA methylation between blood and brain tissue and its application to schizophrenia research. Schizophr Bull.

[28] Tylee DS, Kawaguchi DM, Glatt SJ (2013). On the outside, looking in: a review and evaluation of the comparability of blood and brain “-omes”. Am J Med Genet B Neuropsychiatr Genet.

[29] Lillycrop KA, Costello PM, Teh AL, Murray RJ, Clarke-Harris R, Barton SJ, Garratt ES, Ngo S, Sheppard AM, Wong J (2015). Association between perinatal methylation of the neuronal differentiation regulator HES1 and later childhood neurocognitive function and behaviour. Int J Epidemiol.

[30] Hodyl NA, Roberts CT, Bianco-Miotto T. Cord blood DNA methylation biomarkers for predicting neurodevelopmental outcomes. Genes (Basel). 2016;7(12):117. 10.3390/genes7120117 PMC5192493 27918480

[31] Nemoda Z, Massart R, Suderman M, Hallett M, Li T, Coote M, Cody N, Sun ZS, Soares CN, Turecki G, et al. Maternal depression is associated with DNA methylation changes in cord blood T lymphocytes and adult hippocampi. Transl Psychiatry. 2015;5(4):e545. 10.1038/tp.2015.32 PMC4462598 25849984

[32] Teroganova N, Girshkin L, Suter CM, Green MJ. DNA methylation in peripheral tissue of schizophrenia and bipolar disorder: a systematic review. BMC Genet. 2016;17:27. 10.1186/s12863-016-0332-2 PMC4727379 26809779

[33] Dempster EL, Pidsley R, Schalkwyk LC, Owens S, Georgiades A, Kane F, Kalidindi S, Picchioni M, Kravariti E, Toulopoulou T (2011). Disease-associated epigenetic changes in monozygotic twins discordant for schizophrenia and bipolar disorder. Hum Mol Genet.

[34] Di Francesco A, Arosio B, Falconi A, Micioni Di Bonaventura MV, Karimi M, Mari D, Casati M, Maccarrone M, D’Addario C (2015). Global changes in DNA methylation in Alzheimer’s disease peripheral blood mononuclear cells. Brain Behav Immun.

[35] Aberg KA, Xie LY, McClay JL, Nerella S, Vunck S, Snider S, Beardsley PM, van den Oord EJCG (2013). Testing two models describing how methylome-wide studies in blood are informative for psychiatric conditions. Epigenomics.

[36] Gordon L, Joo JHE, Andronikos R, Ollikainen M, Wallace EM, Umstad MP, Permezel M, Oshlack A, Morley R, Carlin JB (2011). Expression discordance of monozygotic twins at birth effect of intrauterine environment and a possible mechanism for fetal programming. Epigenetics.

[37] Gordon L, Joo JE, Powel JE, Ollikainen M, Novakovic B, Li X, Andronikos R, Cruickshank MN, Conneely KN, Smith AK (2012). Neonatal DNA methylation profile in human twins is specified by a complex interplay between intrauterine environmental and genetic factors, subject to tissue-specific influence. Genome Res.

[38] Kadhim H, SÉBire G (2002). Immune mechanisms in the pathogenesis of cerebral palsy: implication of proinflammatory cytokines and T lymphocytes. Eur J Paediatr Neurol.

[39] Marbini A, Ferrari A, Cioni G, Bellanova MF, Fusco C, Gemignani F (2002). Immunohistochemical study of muscle biopsy in children with cerebral palsy. Brain Dev.

[40] Girard S, Kadhim H, Roy M, Lavoie K, Brochu ME, Larouche A, Sebire G (2009). Role of perinatal inflammation in cerebral palsy. Pediatr Neurol.

[41] Fleiss B, Gressens P (2012). Tertiary mechanisms of brain damage: a new hope for treatment of cerebral palsy?. Lancet Neurol.

[42] Kang M, Min K, Jang J, Kim SC, Kang MS, Jang SJ, Lee JY, Kim SH, Kim MK, An SA, Kim M (2015). Involvement of immune responses in the efficacy of cord blood cell therapy for cerebral palsy. Stem Cells Dev.

[43] Aryee MJ, Jaffe AE, Corrada-Bravo H, Ladd-Acosta C, Feinberg AP, Hansen KD, Irizarry RA (2014). Minfi: a flexible and comprehensive Bioconductor package for the analysis of Infinium DNA methylation microarrays. Bioinformatics.

[44] Chen YA, Lemire M, Choufani S, Butcher DT, Grafodatskaya D, Zanke BW, Gallinger S, Hudson TJ, Weksberg R (2013). Discovery of cross-reactive probes and polymorphic CpGs in the Illumina Infinium HumanMethylation450 microarray. Epigenetics.

[45] Houseman EA, Accomando WP, Koestler DC, Christensen BC, Marsit CJ, Nelson HH, Wiencke JK, Kelsey KT (2012). DNA methylation arrays as surrogate measures of cell mixture distribution. BMC Bioinformatics.

[46] Jaffe AE, Irizarry RA. Accounting for cellular heterogeneity is critical in epigenome-wide association studies. Genome Biol. 2014;15(2): R31. 10.1186/gb-2014-15-2-r31 PMC4053810 24495553

[47] Maksimovic J, Gordon L, Oshlack A (2012). SWAN: subset-quantile within array normalization for illumina infinium HumanMethylation450 BeadChips. Genome Biol.

[48] Maksimovic J, Gagnon-Bartsch JA, Speed TP, Oshlack A (2015). Removing unwanted variation in a differential methylation analysis of Illumina HumanMethylation450 array data. Nucleic Acids Res.

[49] Phipson B, Maksimovic J, Oshlack A (2016). missMethyl: an R package for analyzing data from Illumina’s HumanMethylation450 platform. Bioinformatics.

[50] Hochberg YBY (1995). Controlling the false discovery rate: a practical and powerful approach to multiple testing.

[51] Murphy TM, Wong CC, Arseneault L, Burrage J, Macdonald R, Hannon E, Fisher HL, Ambler A, Moffitt TE, Caspi A, Mill J (2015). Methylomic markers of persistent childhood asthma: a longitudinal study of asthma-discordant monozygotic twins. Clin Epigenetics.

[52] Jaffe AE, Murakami P, Lee H, Leek JT, Fallin MD, Feinberg AP, Irizarry RA (2012). Bump hunting to identify differentially methylated regions in epigenetic epidemiology studies. Int J Epidemiol.

[53] Suchiman HE, Slieker RC, Kremer D, Slagboom PE, Heijmans BT, Tobi EW (2015). Design, measurement and processing of region-specific DNA methylation assays: the mass spectrometry-based method EpiTYPER. Front Genet.

[54] Ollikainen M, Smith KR, Joo EJ, Ng HK, Andronikos R, Novakovic B, Abdul Aziz NK, Carlin JB, Morley R, Saffery R, Craig JM (2010). DNA methylation analysis of multiple tissues from newborn twins reveals both genetic and intrauterine components to variation in the human neonatal epigenome. Hum Mol Genet.

[55] Cruickshank MN, Oshlack A, Theda C, Davis PG, Martino D, Sheehan P, Dai Y, Saffery R, Doyle LW, Craig JM. Analysis of epigenetic changes in survivors of preterm birth reveals the effect of gestational age and evidence for a long term legacy. Genome Med. 2013;5(10):96. 10.1186/gm500 PMC3978871 24134860

[56] Lemire M, Zaidi SH, Zanke BW, Gallinger S, Hudson TJ, Cleary SP (2015). The effect of 5-fluorouracil/leucovorin chemotherapy on CpG methylation, or the confounding role of leukocyte heterogeneity: an illustration. Genomics.

[57] Li Yim AYF, Duijvis NW, Zhao J, J de Jonge W, D’Haens G, Mannens M, Mul A, A te Velde A, Henneman P. Peripheral blood methylation profiling of female Crohn’s disease patients. Clin Epigenetics. 2016;8:65. 10.1186/s13148-016-0230-5 PMC4897922 27279921

[58] Kaut O, Schmitt I, Tost J, Busato F, Liu Y, Hofmann P, Witt SH, Rietschel M, Frohlich H, Wullner U. Epigenome-wide DNA methylation analysis in siblings and monozygotic twins discordant for sporadic Parkinson’s disease revealed different epigenetic patterns in peripheral blood mononuclear cells. Neurogenetics. 2016;18:7–22. 10.1007/s10048-016-0497-x 27709425

[59] Wong CC, Meaburn EL, Ronald A, Price TS, Jeffries AR, Schalkwyk LC, Plomin R, Mill J (2014). Methylomic analysis of monozygotic twins discordant for autism spectrum disorder and related behavioural traits. Mol Psychiatry.

[60] Di H, He Q. The role of inflammatory cytokines in the pathogenesis of cerebral palsy. Gynecol Obstet. 2016;6:360.

[61] Huang L, Sherchan P, Wang Y, Reis C, Applegate RL, Tang J, Zhang JH (2015). Phosphoinositide 3-kinase gamma contributes to neuroinflammation in a rat model of surgical brain injury. J Neurosci.

[62] Smith LR, Meyer G, Lieber RL (2013). Systems analysis of biological networks in skeletal muscle function. Wiley Interdiscip Rev Syst Biol Med.

[63] Jaarsma D, van den Berg R, Wulf PS, van Erp S, Keijzer N, Schlager MA, de Graaff E, De Zeeuw CI, Pasterkamp RJ, Akhmanova A, Hoogenraad CC (2014). A role for Bicaudal-D2 in radial cerebellar granule cell migration. Nat Commun.

[64] Fisher HL, Murphy TM, Arseneault L, Caspi A, Moffitt TE, Viana J, Hannon E, Pidsley R, Burrage J, Dempster EL (2015). Methylomic analysis of monozygotic twins discordant for childhood psychotic symptoms. Epigenetics.

[65] van Dongen J, Nivard MG, Baselmans BM, Zilhao NR, Ligthart L, Consortium B, Heijmans BT, Bartels M, Boomsma DI (2015). Epigenome-wide association study of aggressive behavior. Twin Res Hum Genet.

[66] Nelson KB, Dambrosia JM, Iovannisci DM, Cheng S, Grether JK, Lammer E (2005). Genetic polymorphisms and cerebral palsy in very preterm infants. Pediatr Res.

[67] Gibson CS, MacLennan AH, Dekker GA, Goldwater PN, Sullivan TR, Munroe DJ, Tsang S, Stewart C, Nelson KB (2008). Candidate genes and cerebral palsy: a population-based study. Pediatrics.

[68] Yoon BH, Park CW, Chaiworapongsa T (2003). Intrauterine infection and the development of cerebral palsy. BJOG.

[69] Leviton A. Preterm birth and cerebral palsy: is tumor necrosis factor the missing link? Dev Med Child Neurol. 1993;35:553–558. 10.1111/j.1469-8749.1993.tb11688.x 8504899

[70] Asselbergs FW, Pai JK, Rexrode KM, Hunter DJ, Rimm EB (2007). Effects of lymphotoxin-alpha gene and galectin-2 gene polymorphisms on inflammatory biomarkers, cellular adhesion molecules and risk of coronary heart disease. Clin Sci (Lond).

[71] Wu J, Li X (2015). Plasma tumor necrosis factor-alpha (TNF-α) levels correlate with disease severity in spastic diplegia, triplegia, and quadriplegia in children with cerebral palsy. Med Sci Monit.

[72] Hur EM, Son M, Lee OH, Choi YB, Park C, Lee H, Yun Y (2003). LIME, a novel transmembrane adaptor protein, associates with p56lck and mediates T cell activation. J Exp Med.

[73] Borrego F (2013). The CD300 molecules: an emerging family of regulators of the immune system. Blood.

[74] Kantola AK, Keski-Oja J, Koli K (2005). Induction of human LTBP-3 promoter activity by TGF-beta1 is mediated by Smad3/4 and AP-1 binding elements. Gene.

[75] Kerstin Krieglstein JS, Schober A, Sullivan A, Unsicker K. TGF-Beta and the regulation of neuron survival and death. J Physiol Paris. 2002;96:25–30. 10.1016/s0928-4257(01)00077-8 11755780

[76] Dobolyi A, Vincze C, Pal G, Lovas G (2012). The neuroprotective functions of transforming growth factor beta proteins. Int J Mol Sci.

[77] Hagberg H, Edwards AD, Groenendaal F (2016). Perinatal brain damage: the term infant. Neurobiol Dis.

[78] Malaeb S, Dammann O (2009). Fetal inflammatory response and brain injury in the preterm newborn.

[79] Liu F, Mccullough LD (2013). Inflammatory responses in hypoxic ischemic encephalopathy.

[80] Ho NT, Furge K, Fu W, Busik J, Khoo SK, Lu Q, Lenski M, Wirth J, Hurvitz E, Dodge N (2013). Gene expression in archived newborn blood spots distinguishes infants who will later develop cerebral palsy from matched controls. Pediatr Res.

[81] Shih SC, Claffey KP (1999). Regulation of human vascular endothelial growth factor mRNA stability in hypoxia by heterogeneous nuclear ribonucleoprotein L. J Biol Chem.

[82] Sivakumar V, Ling EA, Lu J, Kaur C (2010). Role of glutamate and its receptors and insulin-like growth factors in hypoxia induced periventricular white matter injury. Glia.

[83] McIntyre S (2015). How low can we go? Recognizing infants at high risk of cerebral palsy earlier.

[84] Suzuki T, Delgado-Escueta AV, Alonso ME, Morita R, Okamura N, Sugimoto Y, Bai D, Medina MT, Bailey JN, Rasmussen A (2006). Mutation analyses of genes on 6p12-p11 in patients with juvenile myoclonic epilepsy. Neurosci Lett.

[85] Afawi Z, Mandelstam S, Korczyn AD, Kivity S, Walid S, Shalata A, Oliver KL, Corbett M, Gecz J, Berkovic SF, Jackson GD (2013). TBC1D24 mutation associated with focal epilepsy, cognitive impairment and a distinctive cerebro-cerebellar malformation. Epilepsy Res.

[86] Ottman R, Annegers JF, Risch N, Hauser WA, Susser M (1996). Relations of genetic and environmental factors in the etiology of epilepsy. Ann Neurol.

[87] Bruck I, Antoniuk SA, Spessatto A, de Bem RS, Hausberger R, Pacheco CG (2001). Epilepsy in children with cerebral palsy. Arq Neuropsiquiatr.

[88] Rakyan VK, Down TA, Balding DJ, Beck S (2011). Epigenome-wide association studies for common human diseases. Nat Rev Genet.

[89] Baque E, Sakzewski L, Barber L, Boyd RN (2016). Systematic review of physiotherapy interventions to improve gross motor capacity and performance in children and adolescents with an acquired brain injury. Brain Inj.

[90] Degerstedt F, Wiklund M, Enberg B (2017). Physiotherapeutic interventions and physical activity for children in Northern Sweden with cerebral palsy: a register study from equity and gender perspectives.

[91] Morgan C, Novak I, Badawi N (2013). Enriched environments and motor outcomes in cerebral palsy: systematic review and meta-analysis. Pediatrics.

[92] Spittle A, Orton J, Anderson PJ, Boyd R, Doyle LW (2015). Early developmental intervention programmes provided posthospital discharge to prevent motor and cognitive impairmentin preterm infants.

[93] Morgan CDJ, Gordon AM, Harbourne R, Spittle A, Johnson R, Fetters L (2016). Effectiveness of motor interventions in infants with cerebralpalsy: a systematic review.

[94] Jacques SM, Qureshi F (1994). Chronic villitis of unknown etiology in twin gestations. Pediatr Pathol.

[95] Wong N, Morley R, Saffery R, Craig J (2008). Archived Guthrie blood spots as a novel source for quantitative DNA methylation analysis. BioTechniques.

[96] Cruickshank MN, Pitt J, Craig JM (2012). Going back to the future with Guthrie-powered epigenome-wide association studies. Genome Med.

[97] Wockner LF, Noble EP, Lawford BR, Young RM, Morris CP, Whitehall VL, Voisey J (2014). Genome-wide DNA methylation analysis of human brain tissue from schizophrenia patients. Transl Psychiatry.

[98] Masliah E, Dumaop W, Galasko D, Desplats P (2013). Distinctive patterns of DNA methylation associated with Parkinson disease: identification of concordant epigenetic changes in brain and peripheral blood leukocytes. Epigenetics.

[99] Horvath S, Zhang Y, Langfelder P, Kahn RS, Boks MP, van Eijk K, van den Berg LH, Ophoff RA (2012). Aging effects on DNA methylation modules in human brain and blood tissue. Genome Biol.

[100] Dempster EL, Wong CC, Lester KJ, Burrage J, Gregory AM, Mill J, Eley TC (2014). Genome-wide methylomic analysis of monozygotic twins discordant for adolescent depression. Biol Psychiatry.

[101] Tsai PC, Bell JT. Power and sample size estimation for epigenome-wide association scans to detect differential DNA methylation. Int J Epidemiol. 2015;44:1429–1441. 10.1093/ije/dyv041 PMC4588864 25972603

[102] Smith AK, Kilaru V, Klengel T, Mercer KB, Bradley B, Conneely KN, Ressler KJ, Binder EB (2015). DNA extracted from saliva for methylation studies of psychiatric traits: evidence for tissue specificity and relatedness to brain. Am J Med Genet B Neuropsychiatr Genet.

[103] Rollins B, Martin MV, Morgan L, Vawter MP (2010). Analysis of whole genome biomarker expression in blood and brain. Am J Med Genet B Neuropsychiatr Genet.

[104] Xin YR, Chanrion B, Liu MM, Galfalvy H, Costa R, Ilievski B, Rosoklija G, Arango V, Dwork AJ, Mann JJ, et al. Genome-wide divergence of DNA methylation marks in cerebral and cerebellar cortices. PLoS One. 2010;5:e11357. 10.1371/journal.pone.0011357 PMC2893206 20596539

[105] Ladd-Acosta C, Pevsner J, Sabunciyan S, Yolken RH, Webster MJ, Dinkins T, Callinan PA, Fan JB, Potash JB, Feinberg AP (2007). DNA methylation signatures within the human brain. Am J Hum Genet.

[106] Hannon E, Lunnon K, Schalkwyk L, Mill J (2015). Interindividual methylomic variation across blood, cortex, and cerebellum: implications for epigenetic studies of neurological and neuropsychiatric phenotypes. Epigenetics.

[107] Kundakovic M, Gudsnuk K, Herbstman JB, Tang D, Perera FP, Champagne FA (2015). DNA methylation of BDNF as a biomarker of early-life adversity. Proc Natl Acad Sci U S A.

[108] Pihlstrom L, Berge V, Rengmark A, Toft M (2015). Parkinson’s disease correlates with promoter methylation in the alpha-synuclein gene. Mov Disord.

[109] Bonellie SR, Currie D, Chalmers J (2005). Comparison of risk factors for cerebral palsy in twins and singletons.

