# Supplementary figures and images for: Epigenome-wide analysis in newborn blood spots from monozygotic twins discordant for cerebral palsy reveals consistent regional differences in DNA methylation
Source: Clin Epigenetics. 2018 Feb 23;10:25. doi: 10.1186/s13148-018-0457-4 (PMC5824607; doi:10.1186/s13148-018-0457-4)

## Slide 1
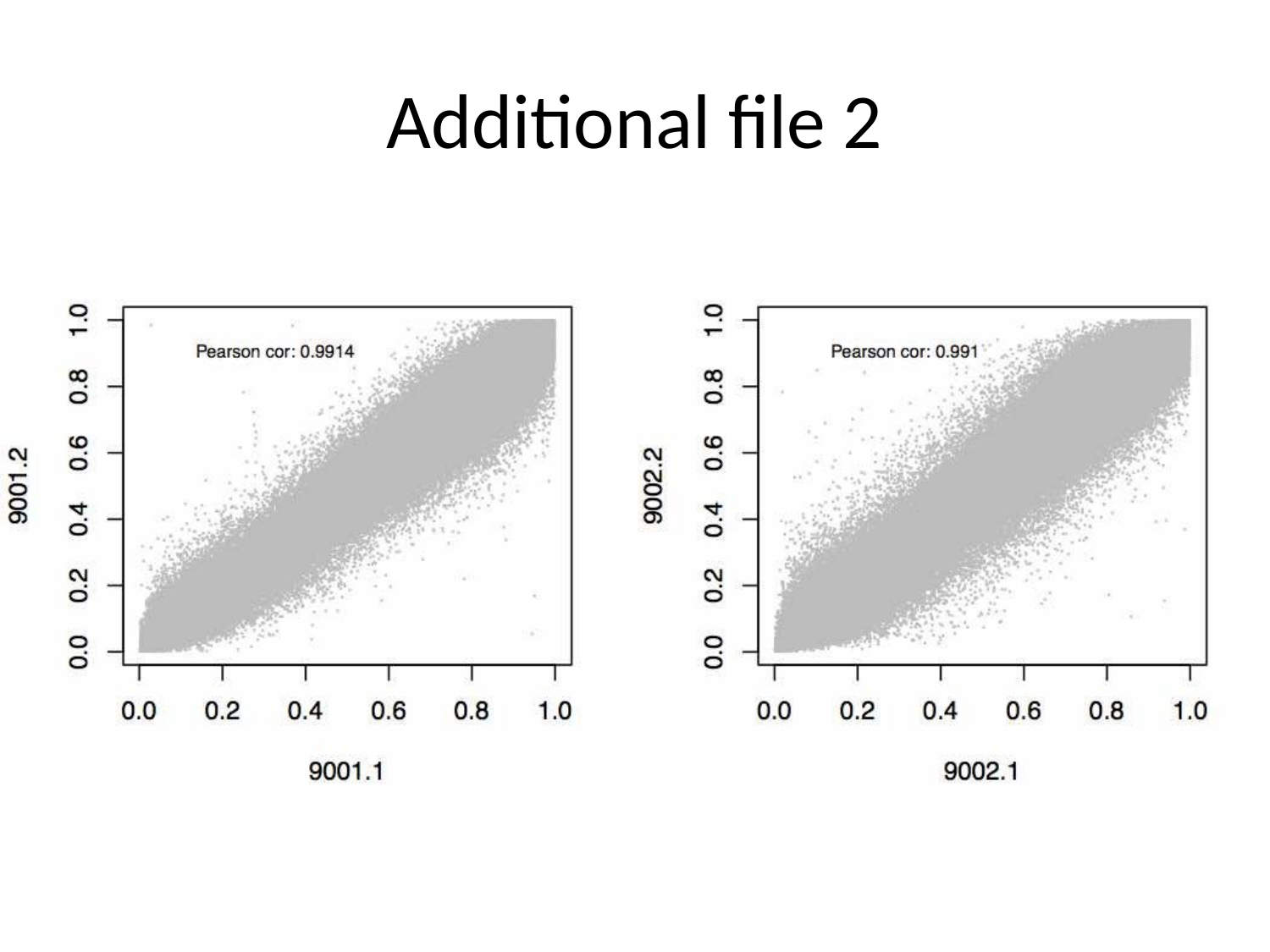

# Additional file 2

## Slide 2
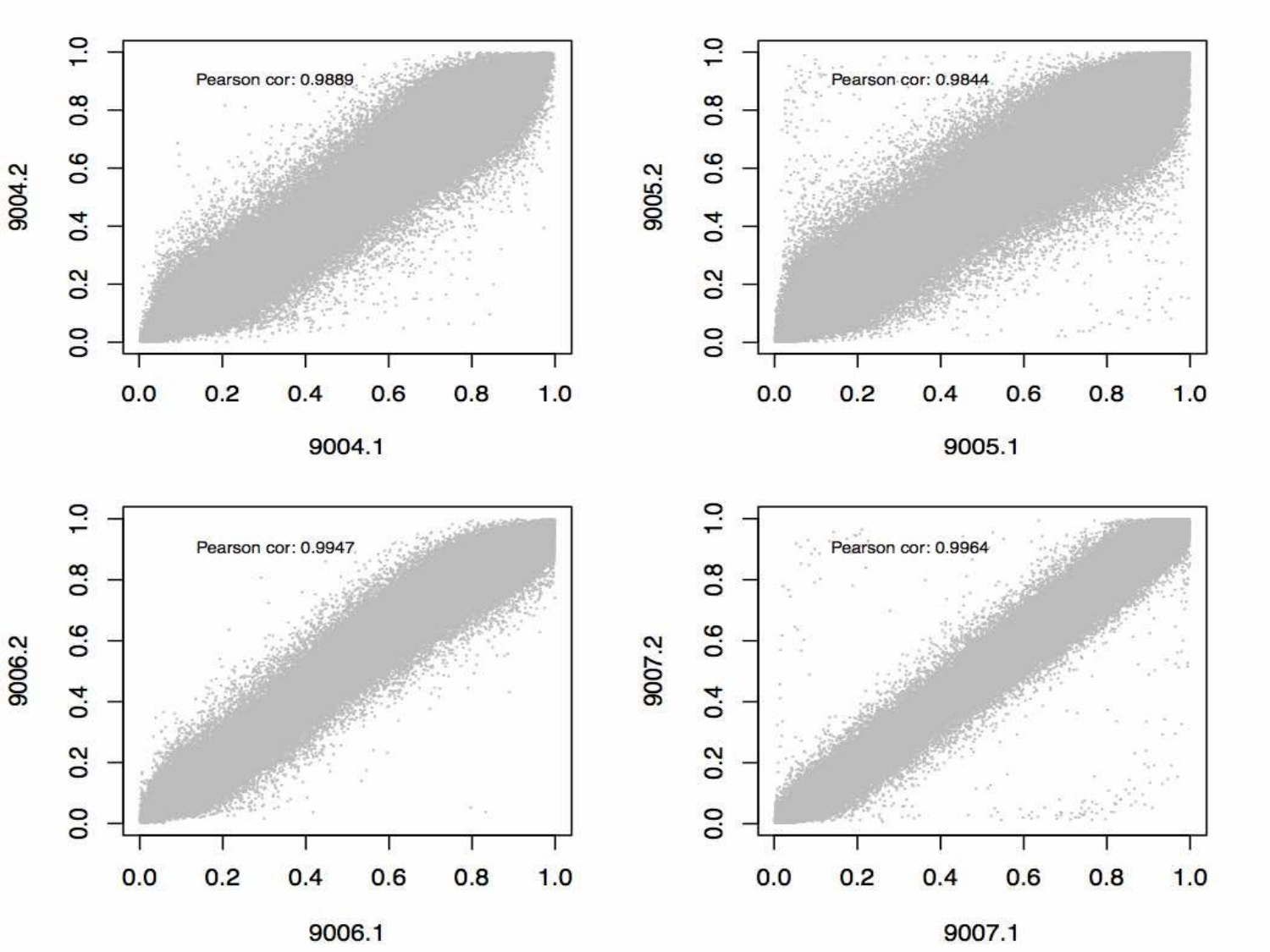

## Slide 3
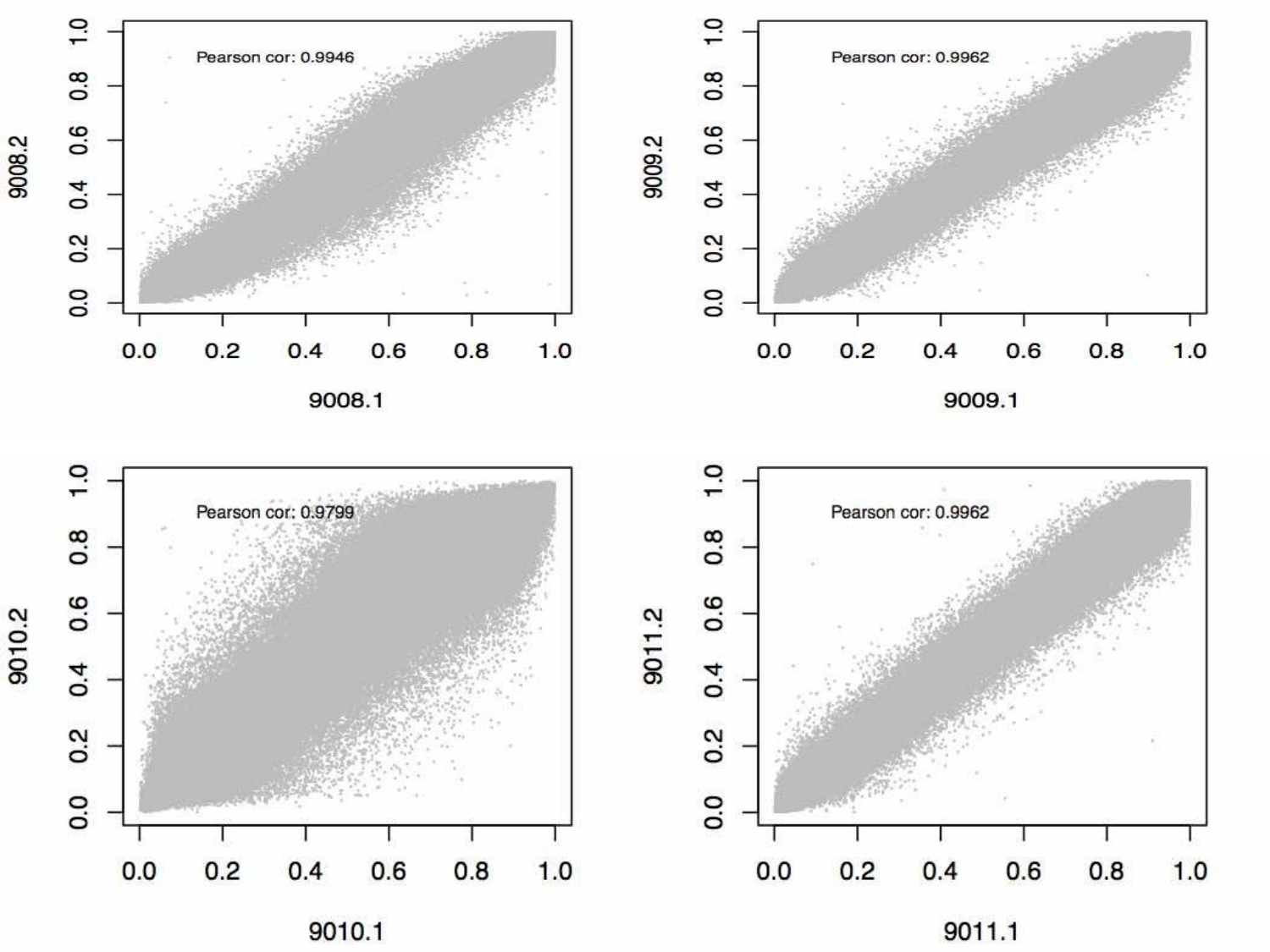

## Slide 4
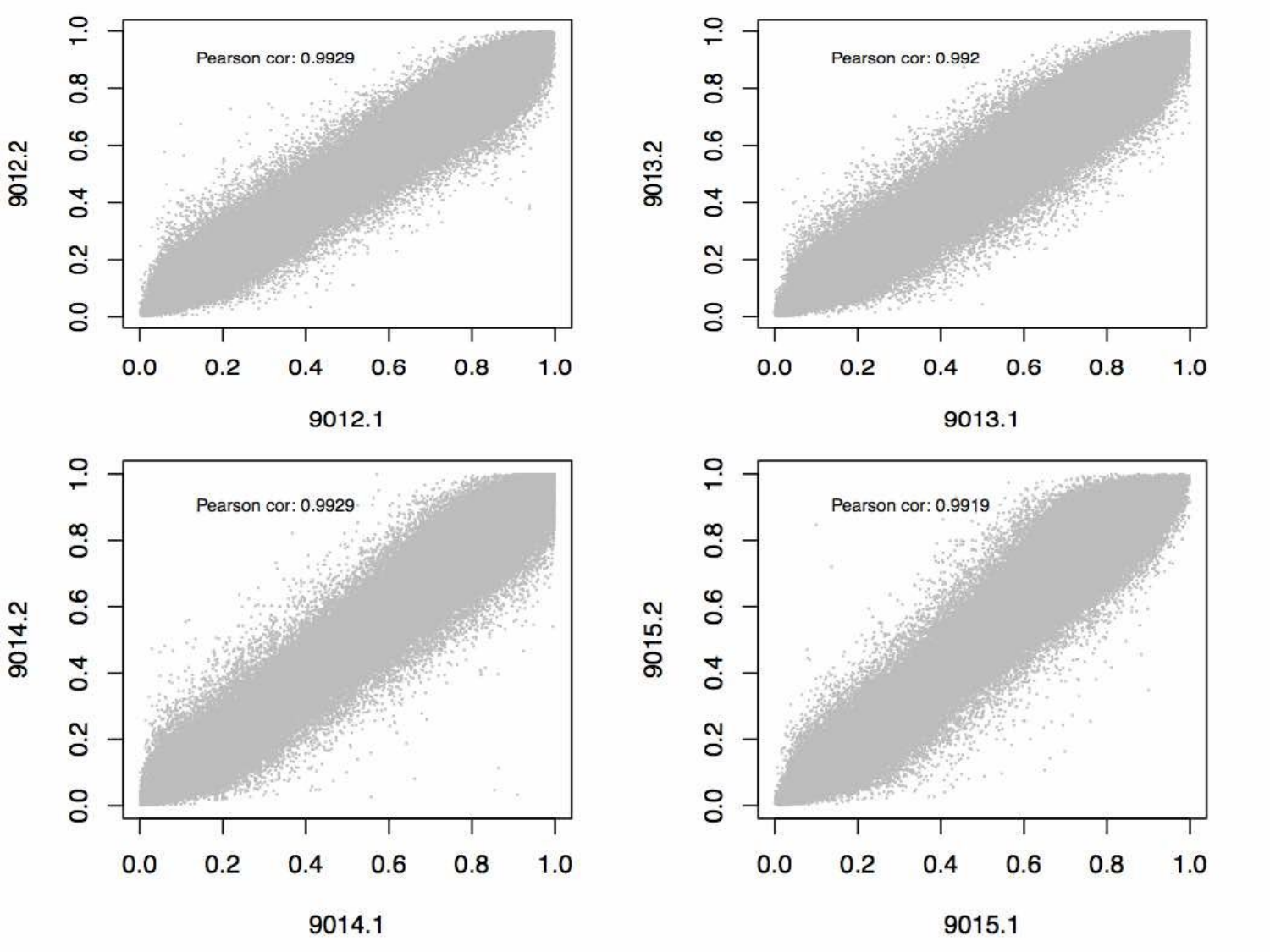

## Slide 5
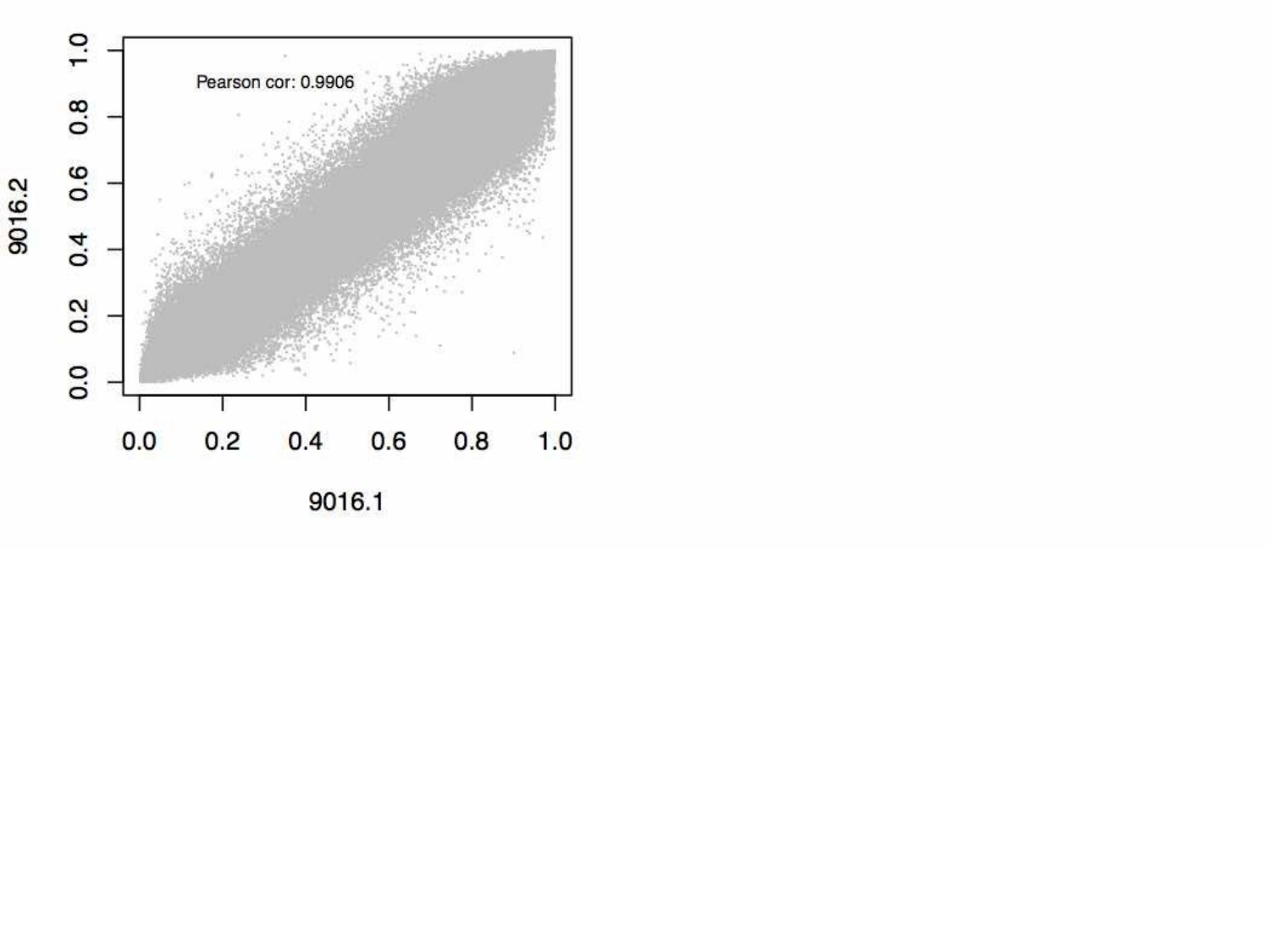

Supplement: Supplementary file 2 — Scatter plots of genome-wide DNA methylation discordance within twin groups. (PPTX 331 kb) [file 13148_2018_457_MOESM2_ESM.pptx]

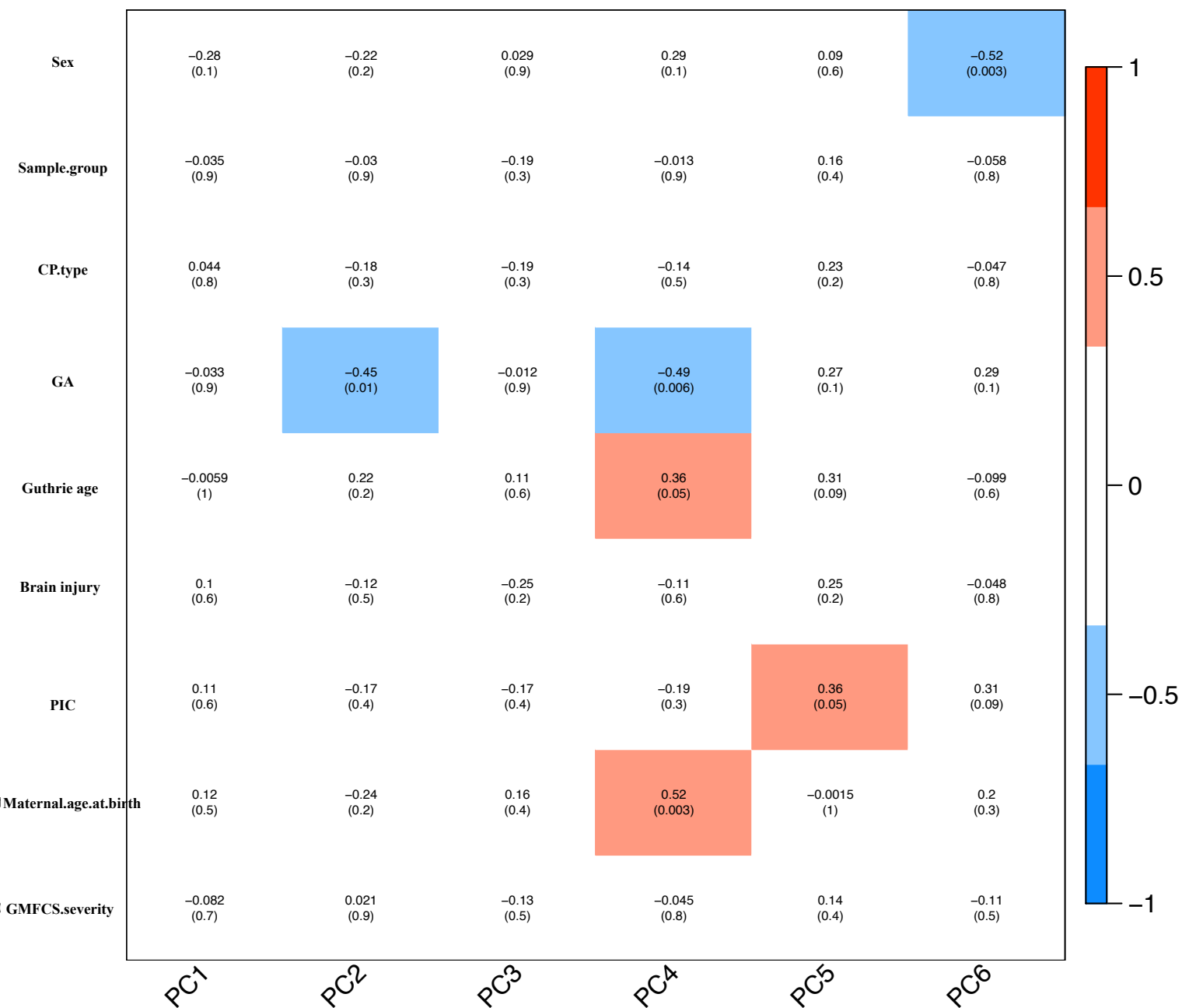

Supplement: Supplementary file 3 — Heat map of the associations between the six largest principal components and specified covariates. The heat map provides a score of the strength of the association between DNA methylation (using M values) and each covariate, with positive and negative correlations ranging according to the magnitude (red positive, blue negative). The values in brackets for each association represent the p-value of the correlation. Of the six significant (p < 0.05) associations, all are weak (correlation < 0.6). Abbreviations: CP, cerebral palsy; PC, principal component; PIC, person in charge of performing DNA extraction; GA, gestational age; GMFCS, gross motor function classification system; Guthrie age, age in postnatal days when Guthrie card was made. (PDF 53 kb) [file 13148_2018_457_MOESM3_ESM.pdf]

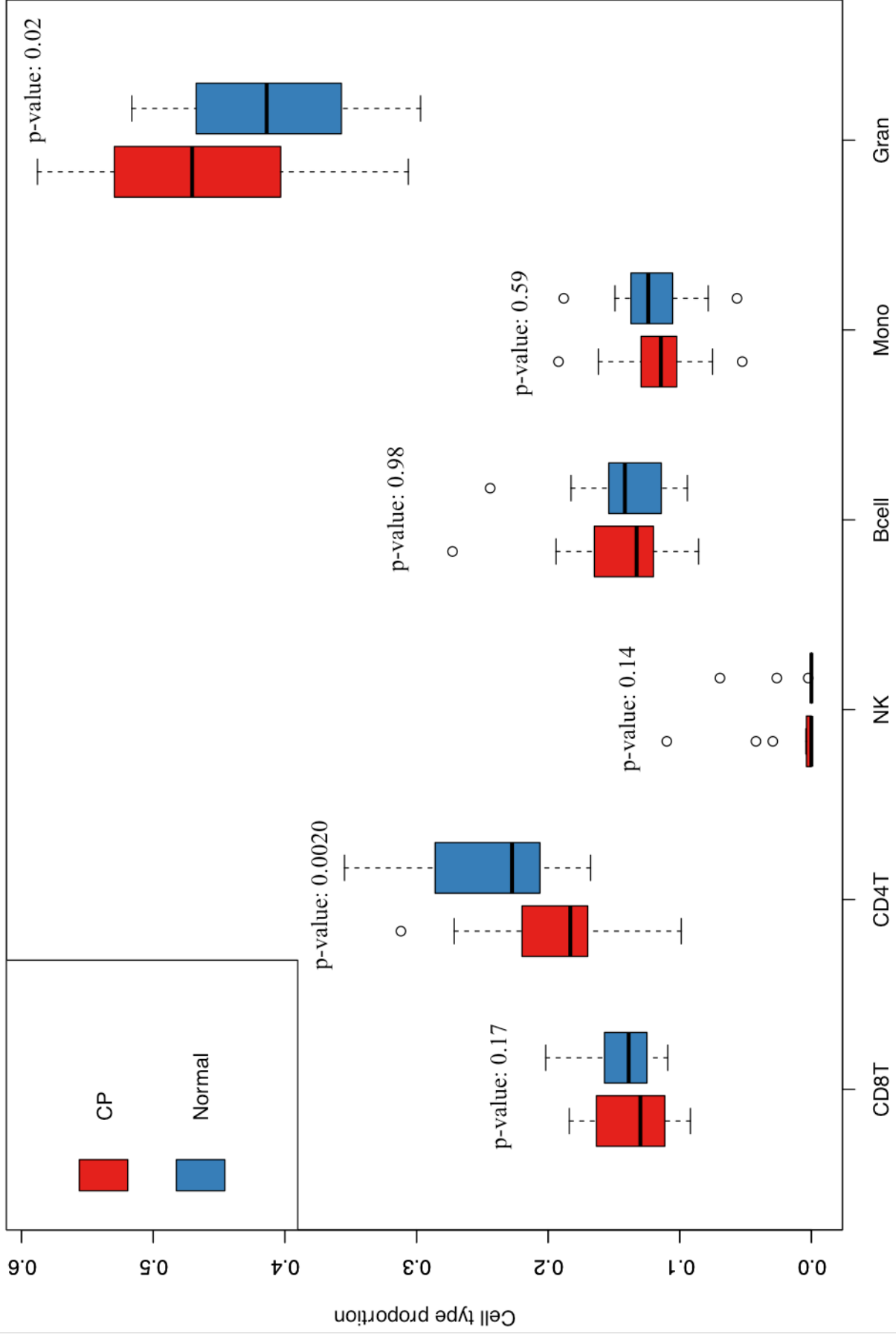

Supplement: Supplementary file 4 — MDS plots for preprocessed data. Samples are coloured based on chip location ranging from 1 to 3. The figure represents similarities between samples’ 1000 most variable probes based on Euclidean distance (sum of squared differences). Dimension 1 represents the largest variation in the dataset, and 2 and 3 are the second and third largest, respectively. (PDF 42 kb) [file 13148_2018_457_MOESM4_ESM.pdf]

***LIME1* - cg24631526**

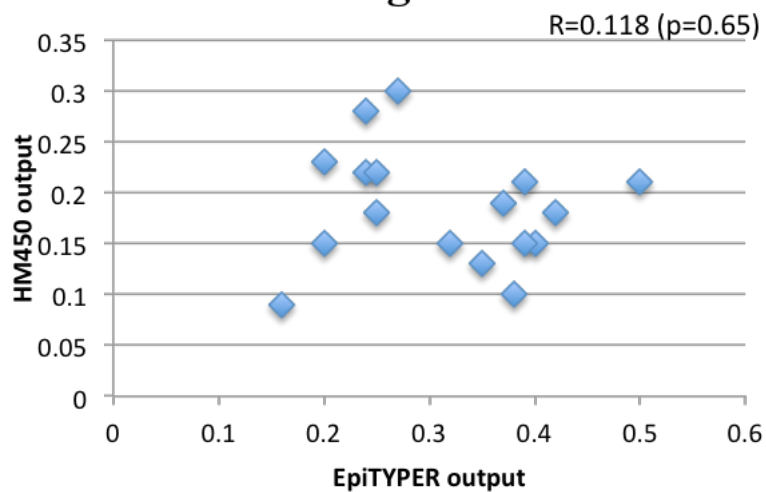

***LIME1* - cg21201401**

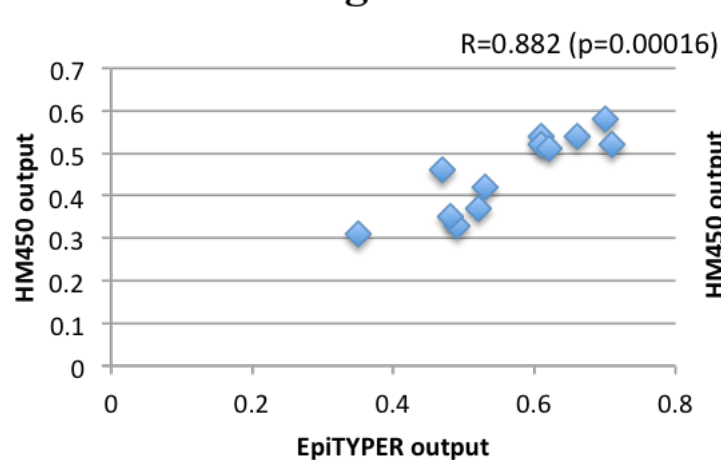

***LIME1* - cg06653796**

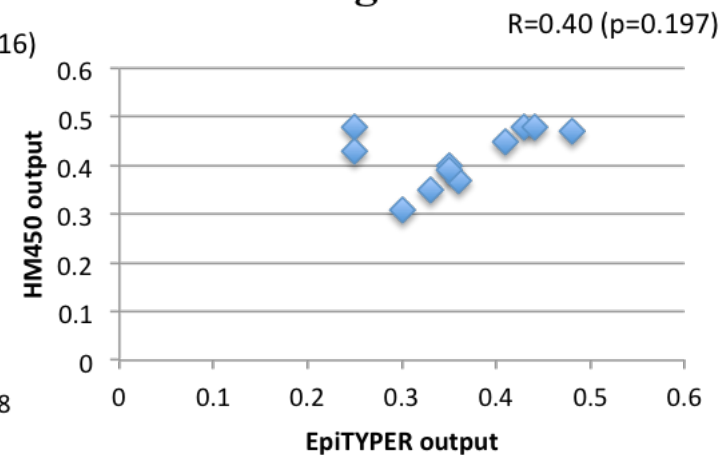

***LTA* - cg14597739/  
cg16219283**

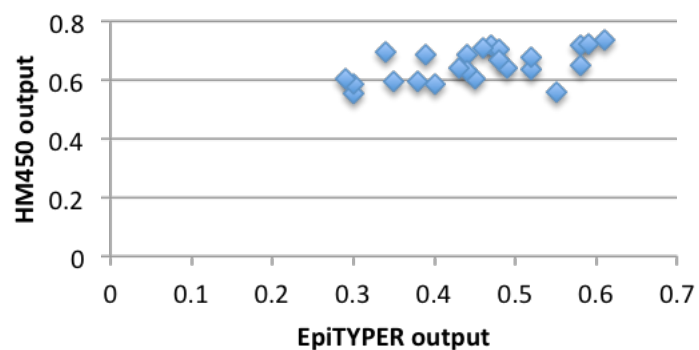

***LTA* - cg21999229**

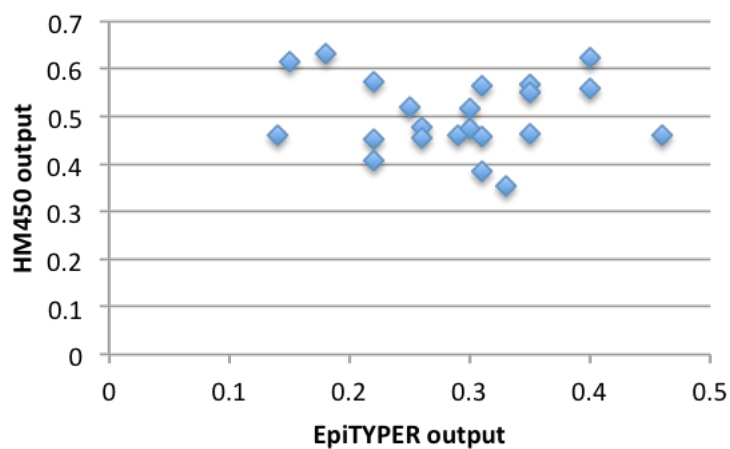

***LTA* - cg11586857**

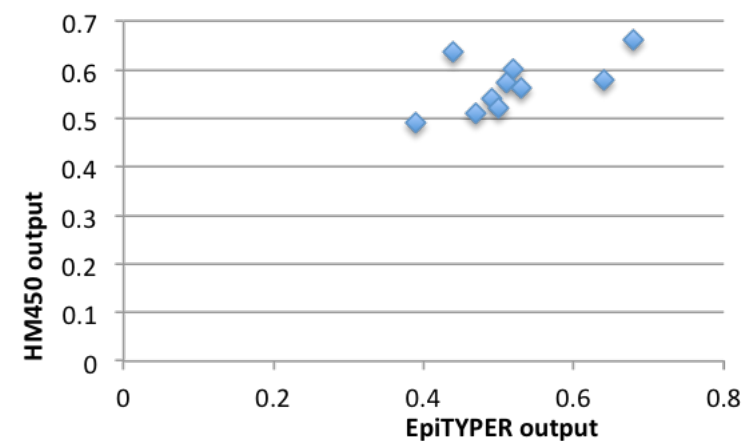

Supplement: Supplementary file 7 — Gene ontology (GO) analysis for top DMRs ranked by p-value (XLSX 38 kb) [file 13148_2018_457_MOESM7_ESM.zip › Additional_file_7.pdf]

## Additional file 11

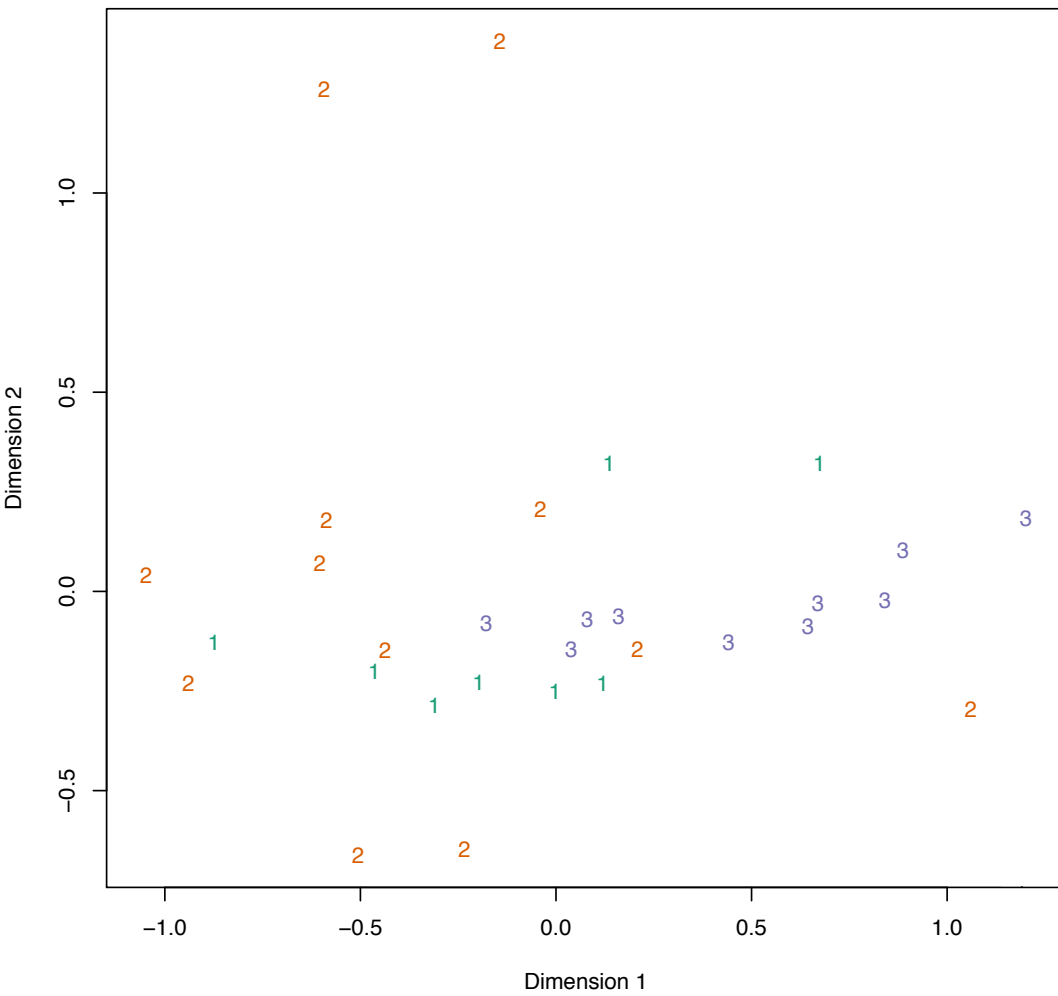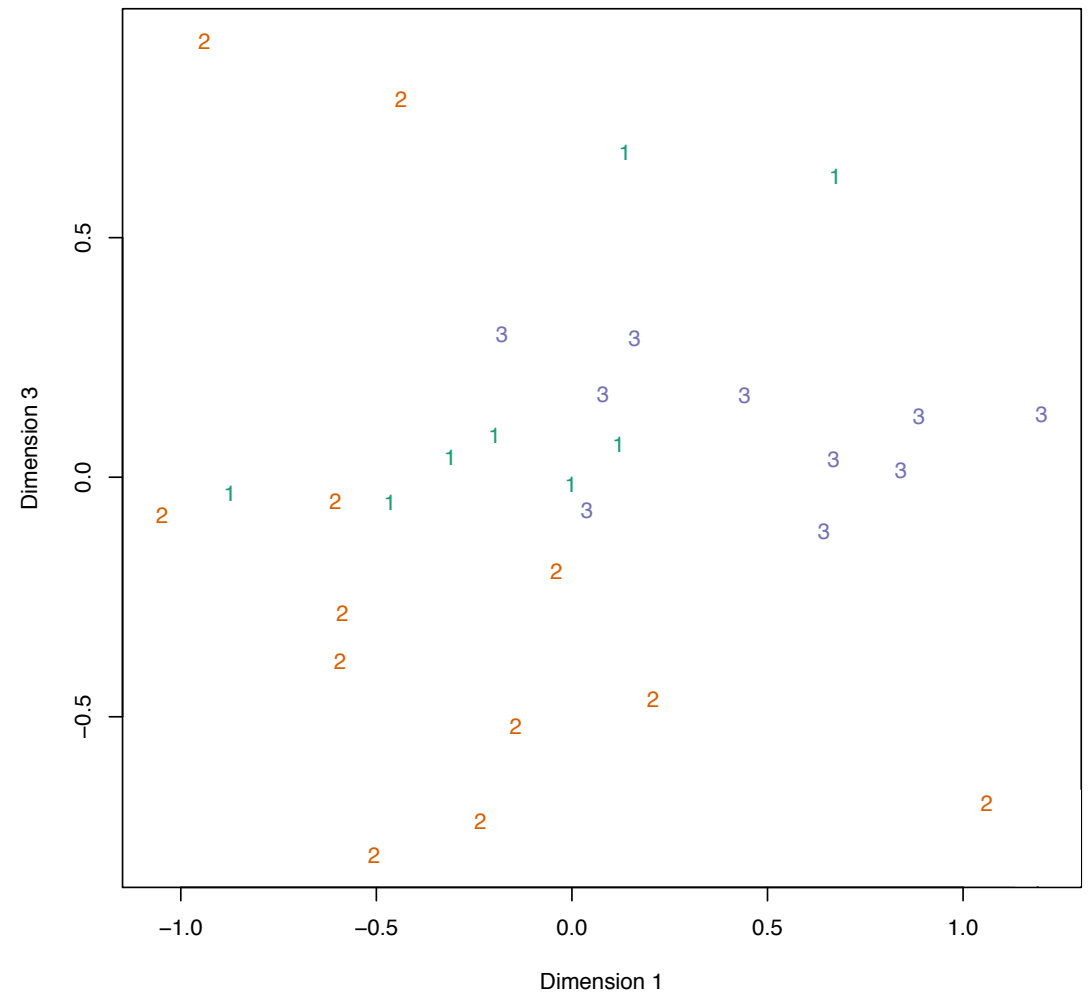

Supplement: Supplementary file 11 — CpG sites (probes) within each twin pair group with an absolute methylation difference > 0.5 and their corresponding genes. Genes are colour coded to highlight overlaps between twin pair groups. (XLSX 30 kb) [file 13148_2018_457_MOESM11_ESM.pdf]
