# Supplementary material for: Epigenome-wide analysis in newborn blood spots from monozygotic twins discordant for cerebral palsy reveals consistent regional differences in DNA methylation
Source: Clin Epigenetics. 2018 Feb 23;10:25. doi: 10.1186/s13148-018-0457-4 (PMC5824607; doi:10.1186/s13148-018-0457-4)
Supplement: Supplementary file 8 — Cross-platform validation of the two top DMRs, LTA and LIME1, between HM450 and EpiTYPER platforms. Pearson’s correlation coefficients for each probe are shown. The scale of both axes reflects a methylation value between 0 and 1 (β). The regression lines are shown in black. Based on the r value (correlation coefficient), correlations across both platforms are shown. The p-value indicates the significance of the correlation. (ZIP 126 kb) [file 13148_2018_457_MOESM8_ESM.pptx]

## Slide 1
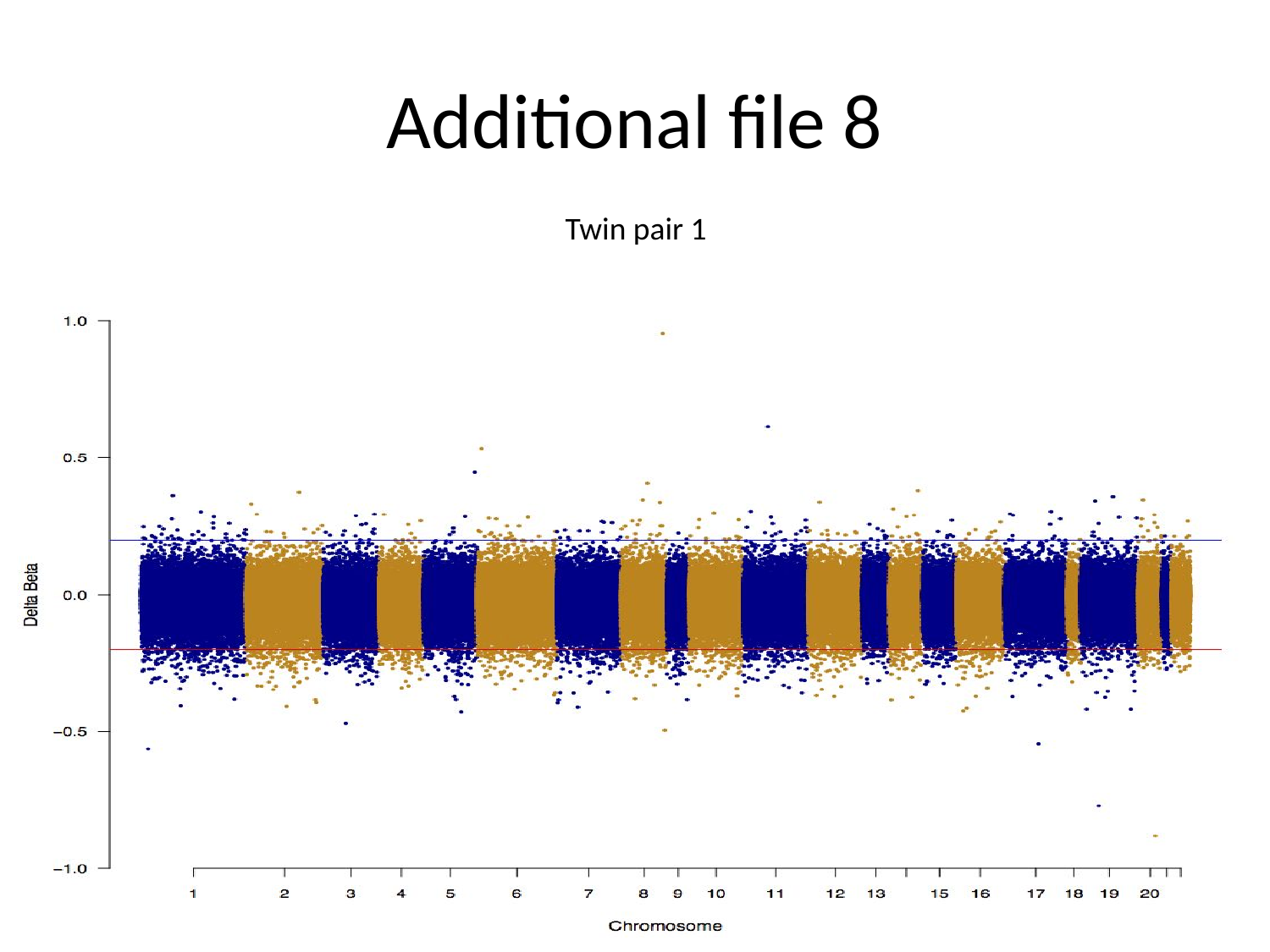

# Additional file 8
Twin pair 1

## Slide 2
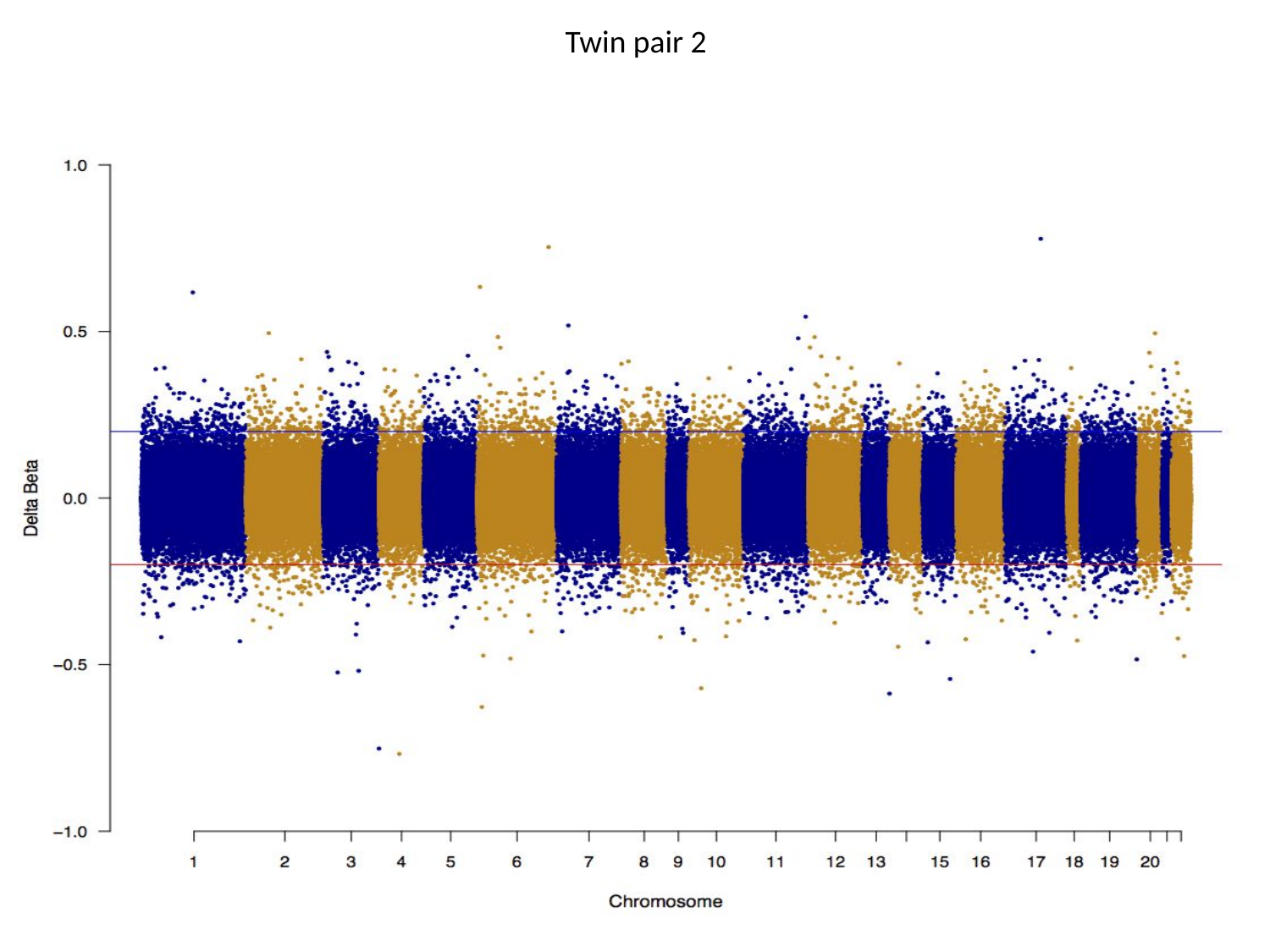

Twin pair 2

## Slide 3
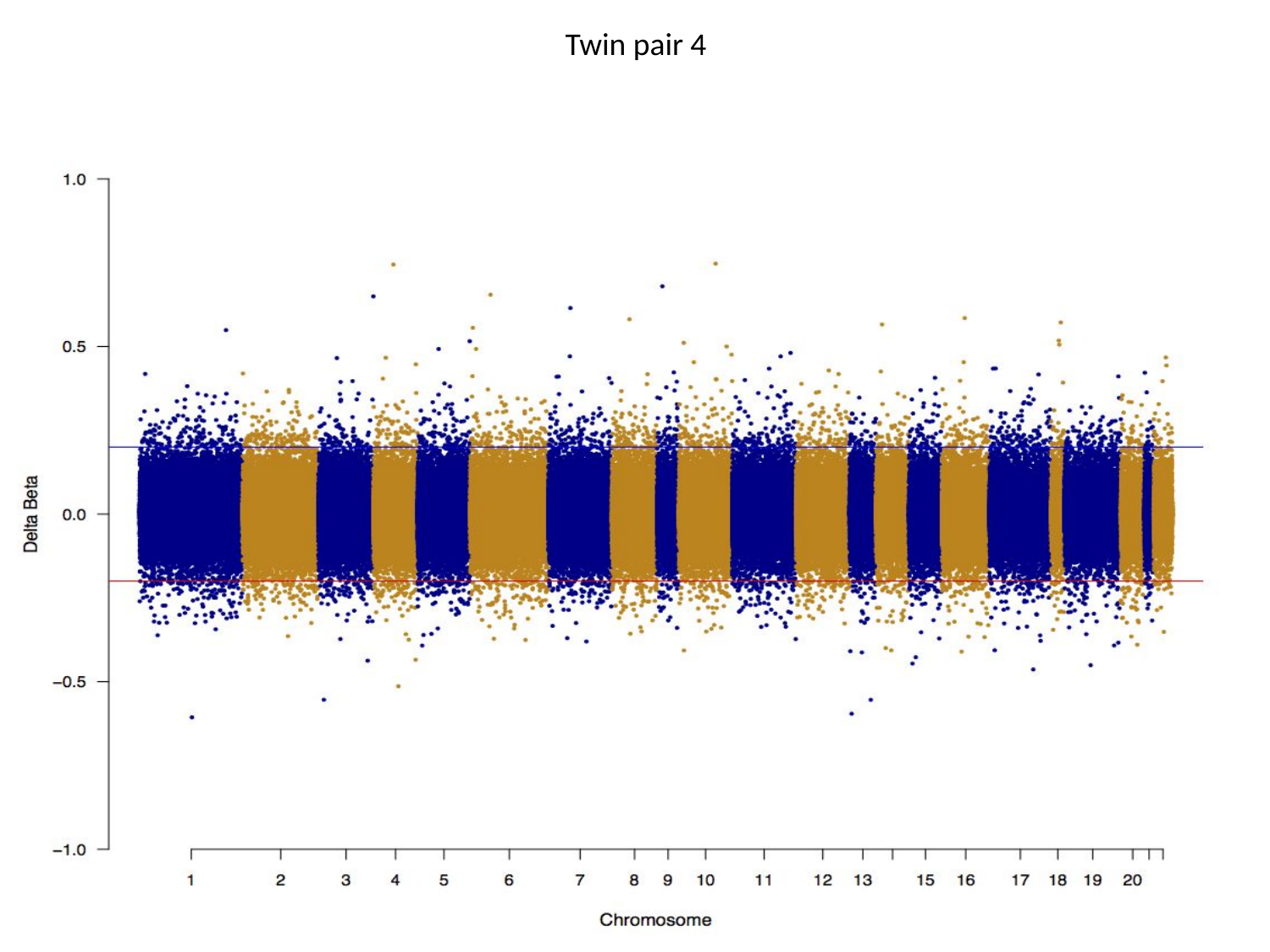

Twin pair 4

## Slide 4
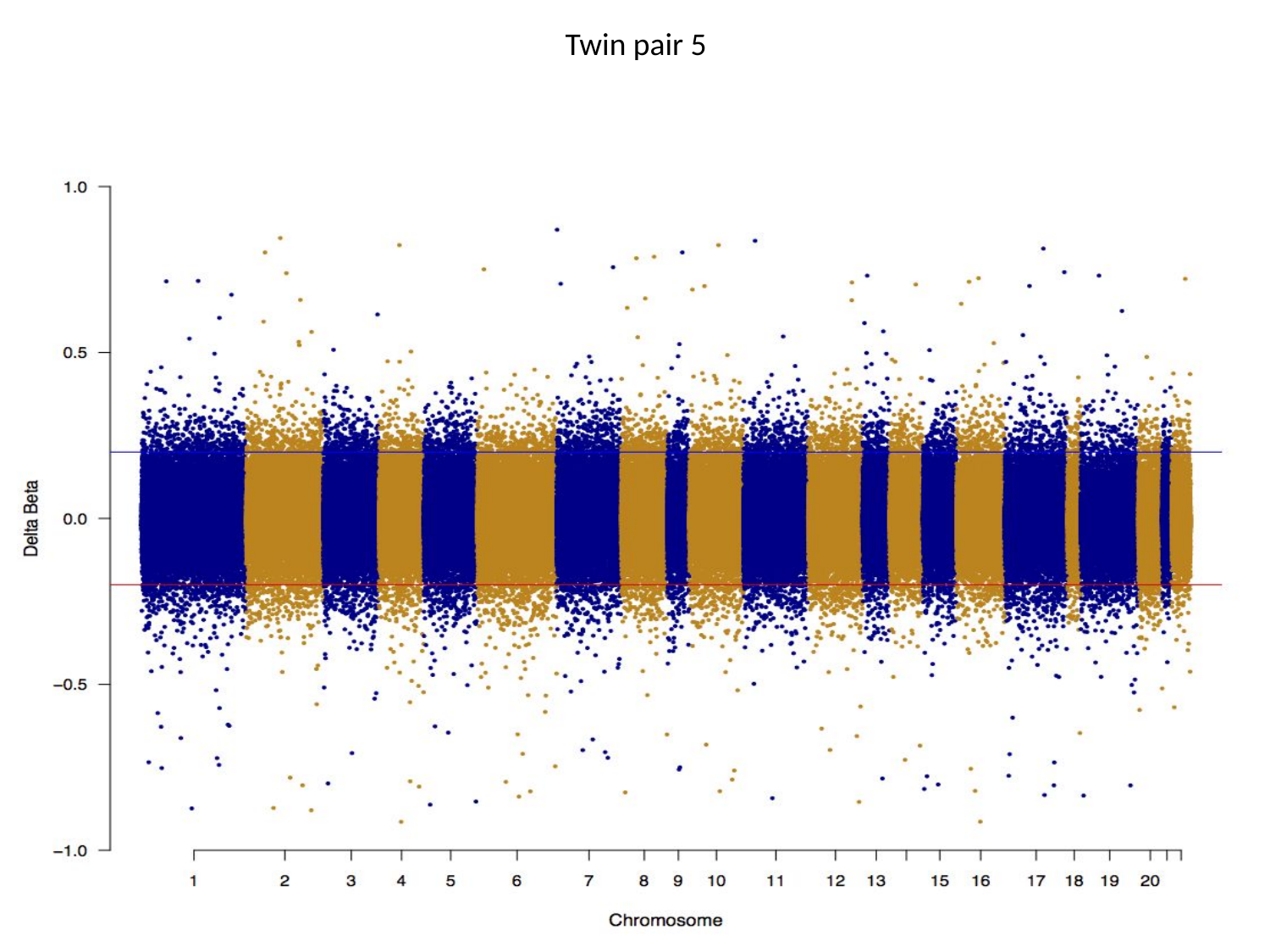

Twin pair 5

## Slide 5
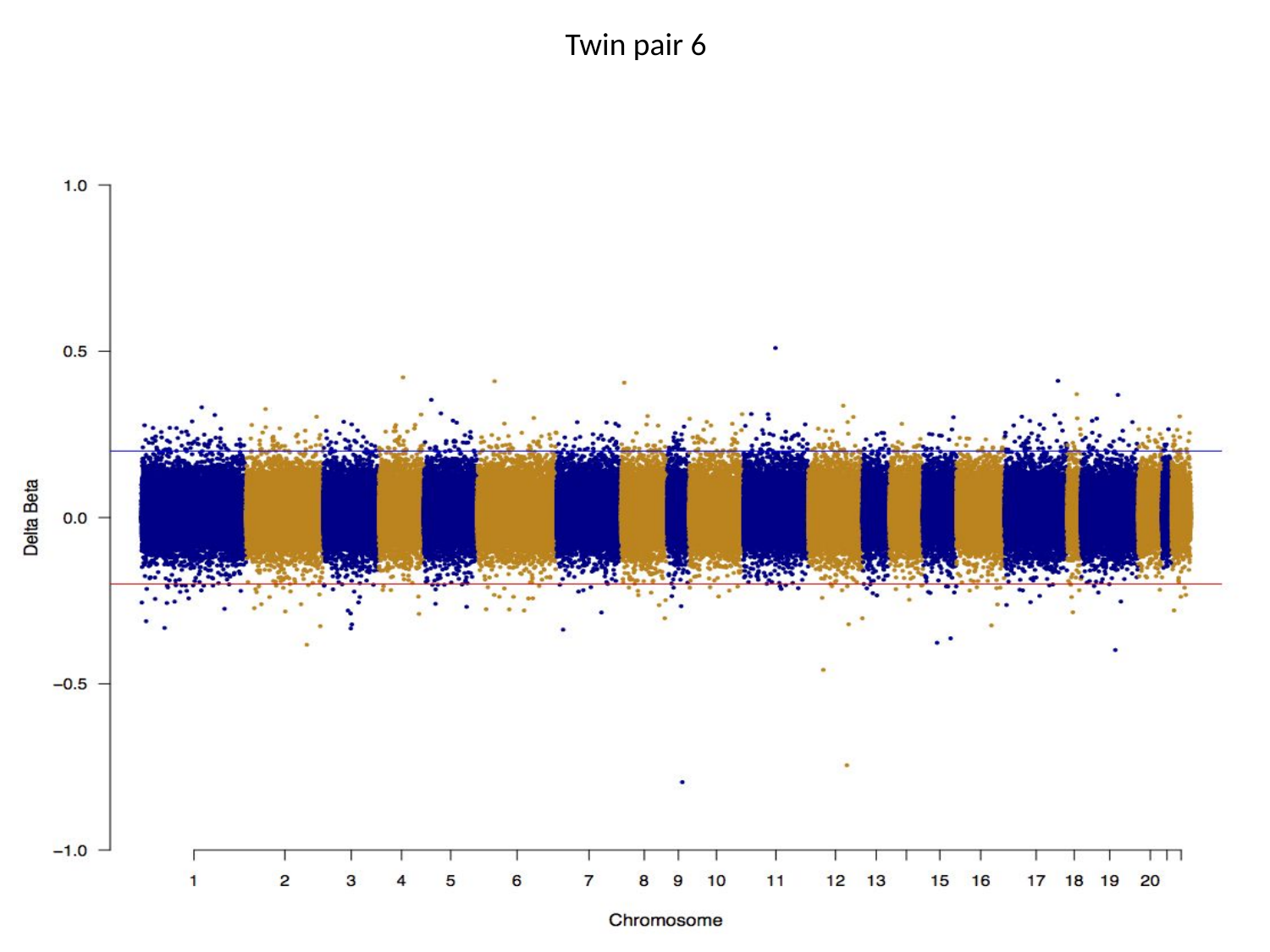

Twin pair 6

## Slide 6
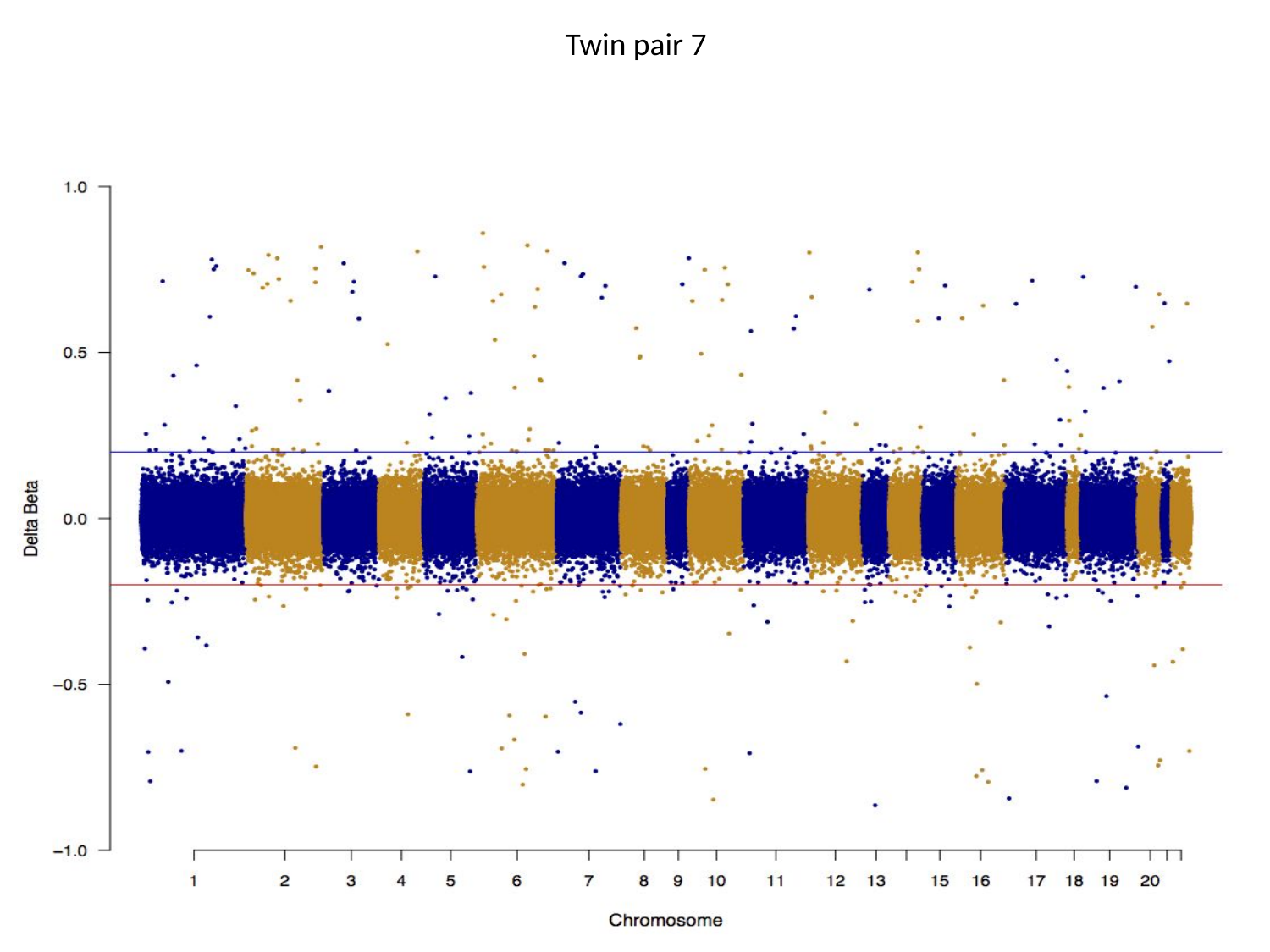

Twin pair 7

## Slide 7
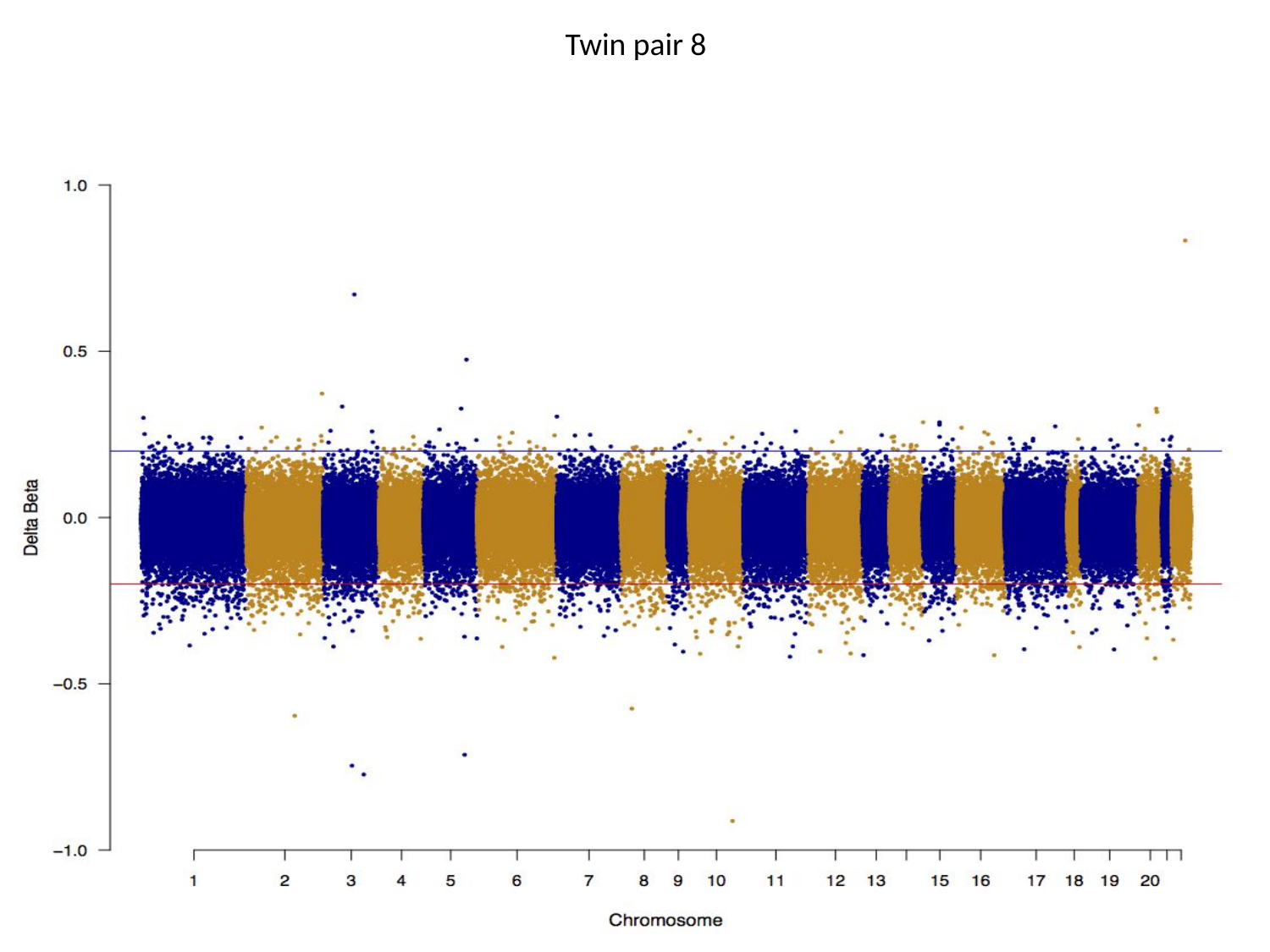

Twin pair 8

## Slide 8
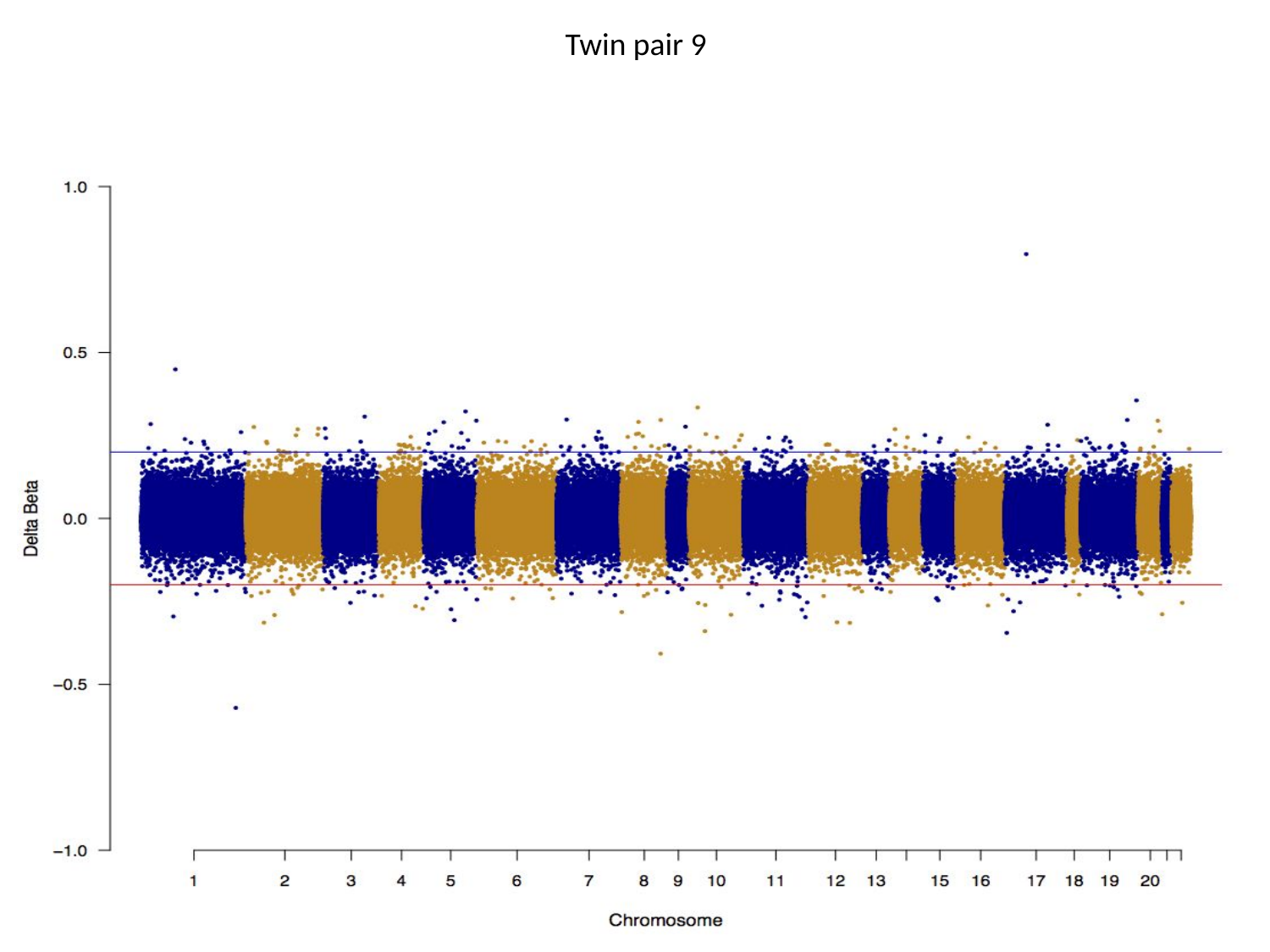

Twin pair 9

## Slide 9
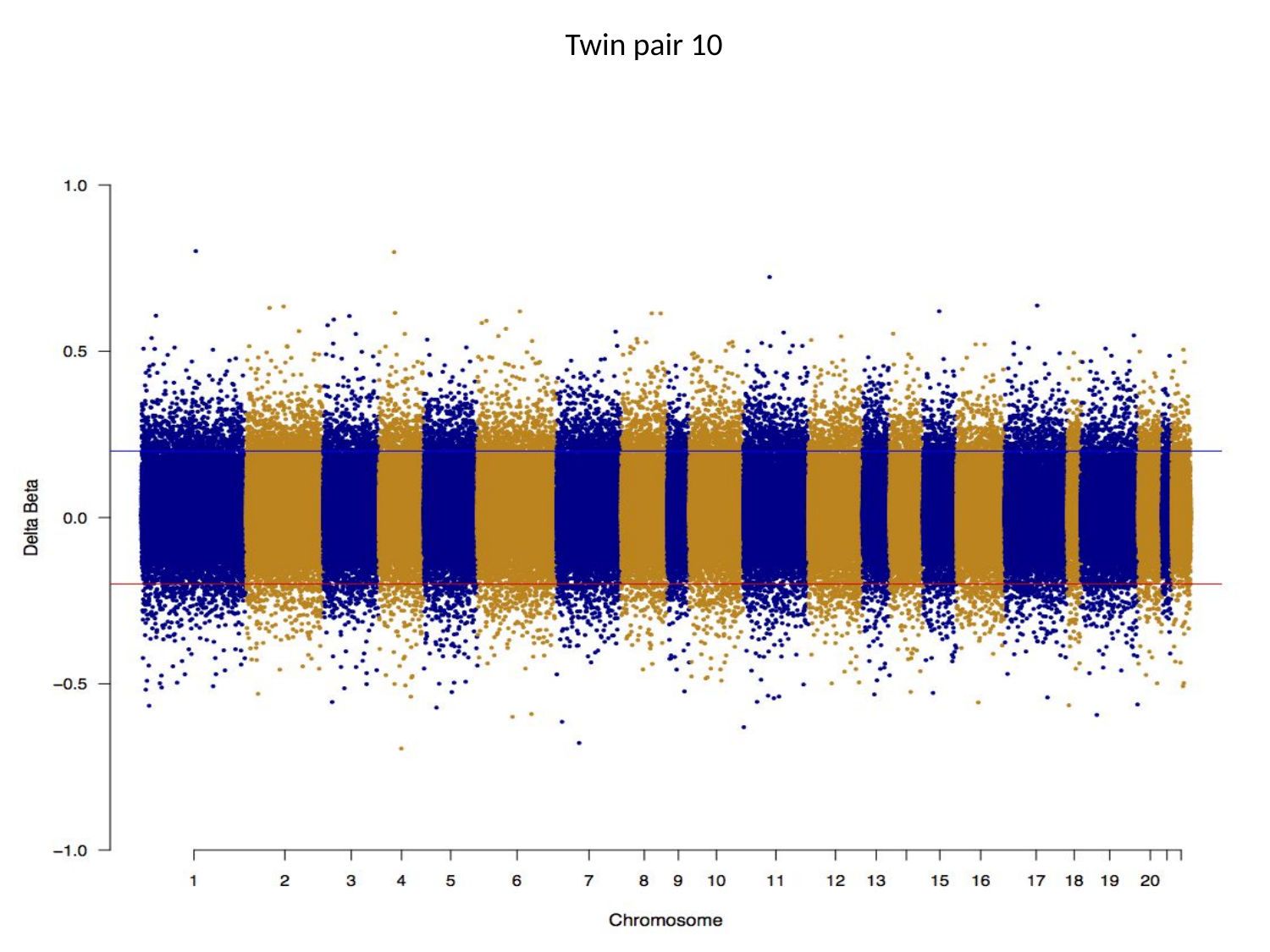

Twin pair 10

## Slide 10
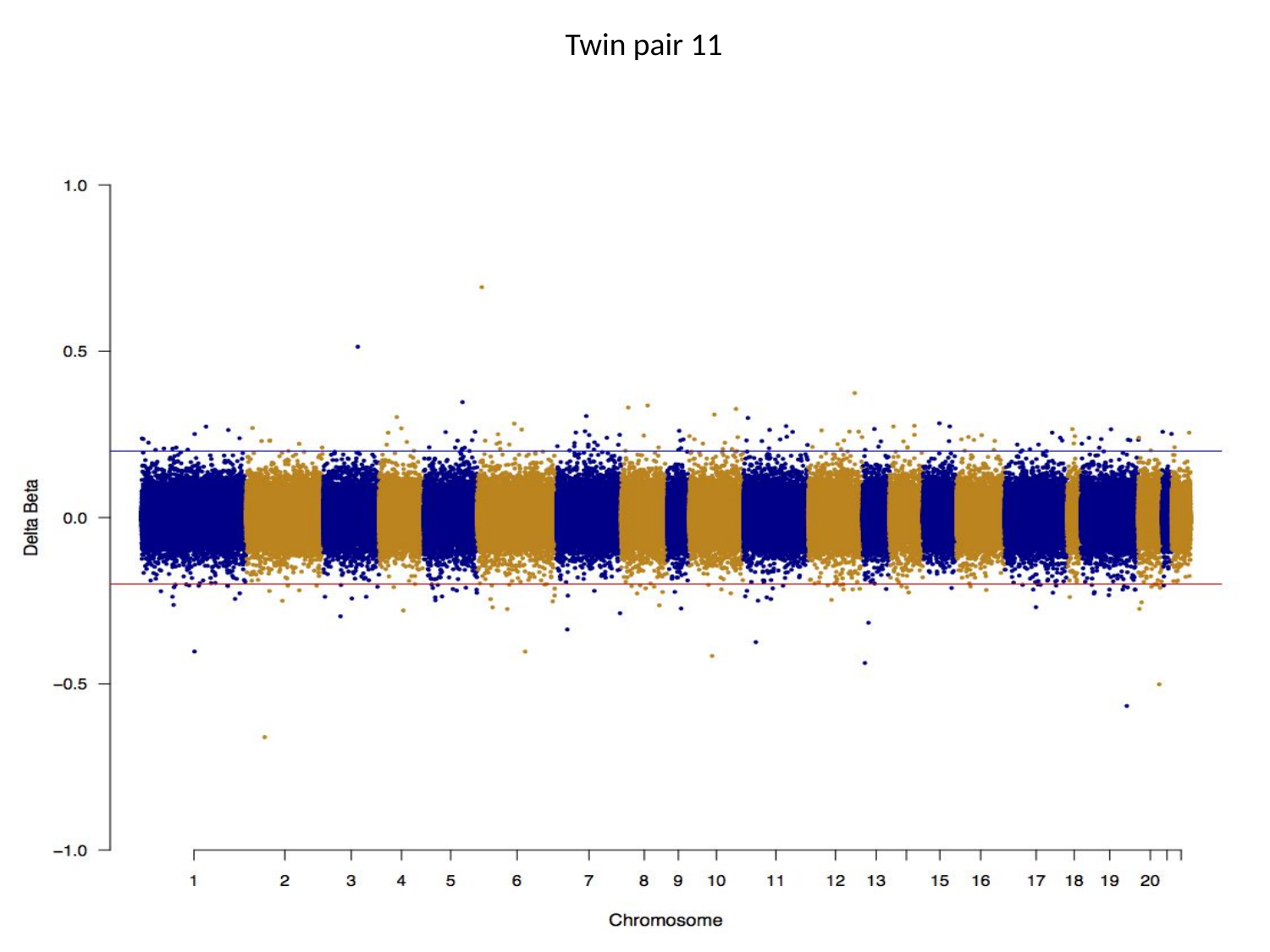

Twin pair 11

## Slide 11
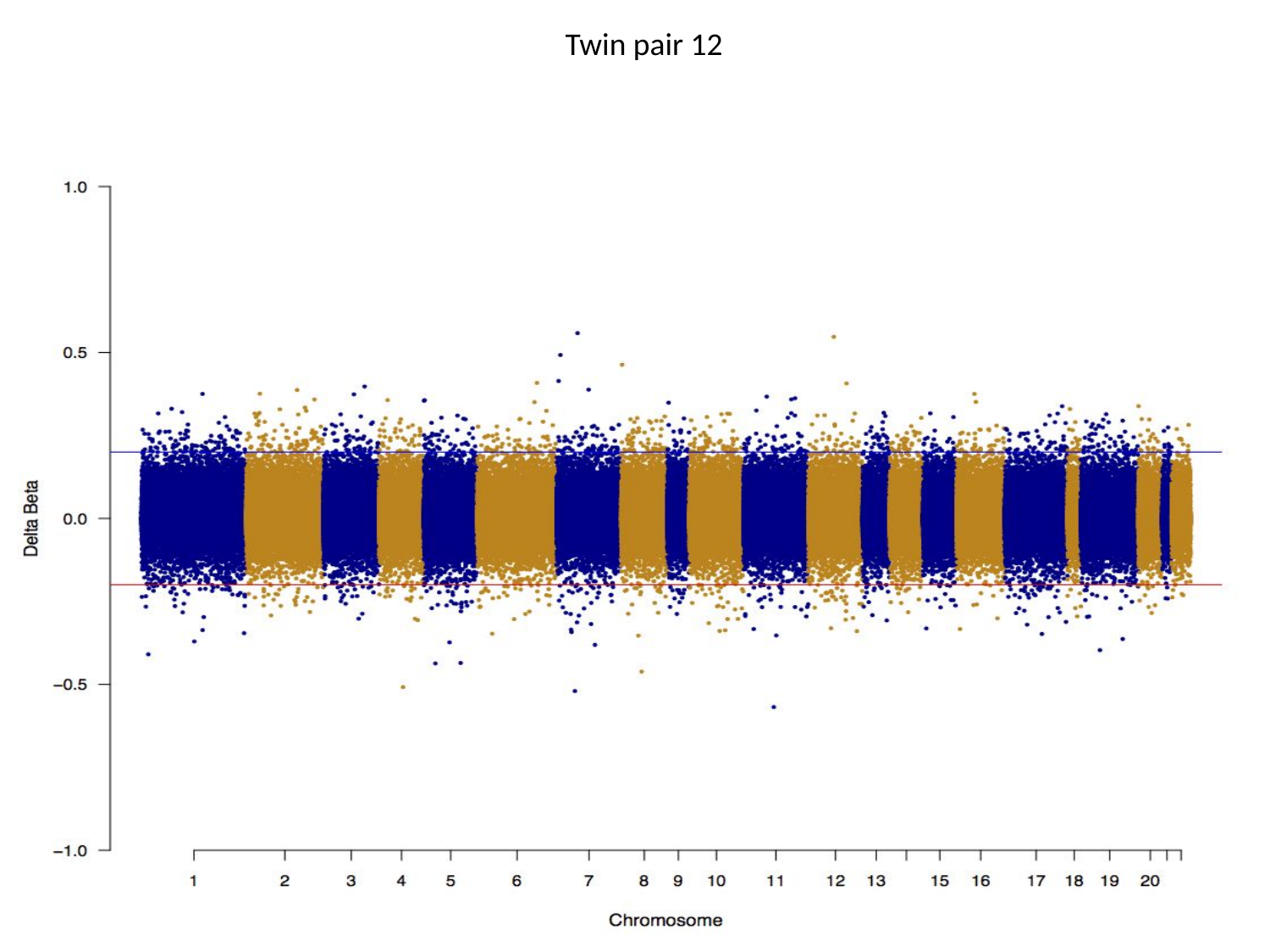

Twin pair 12

## Slide 12
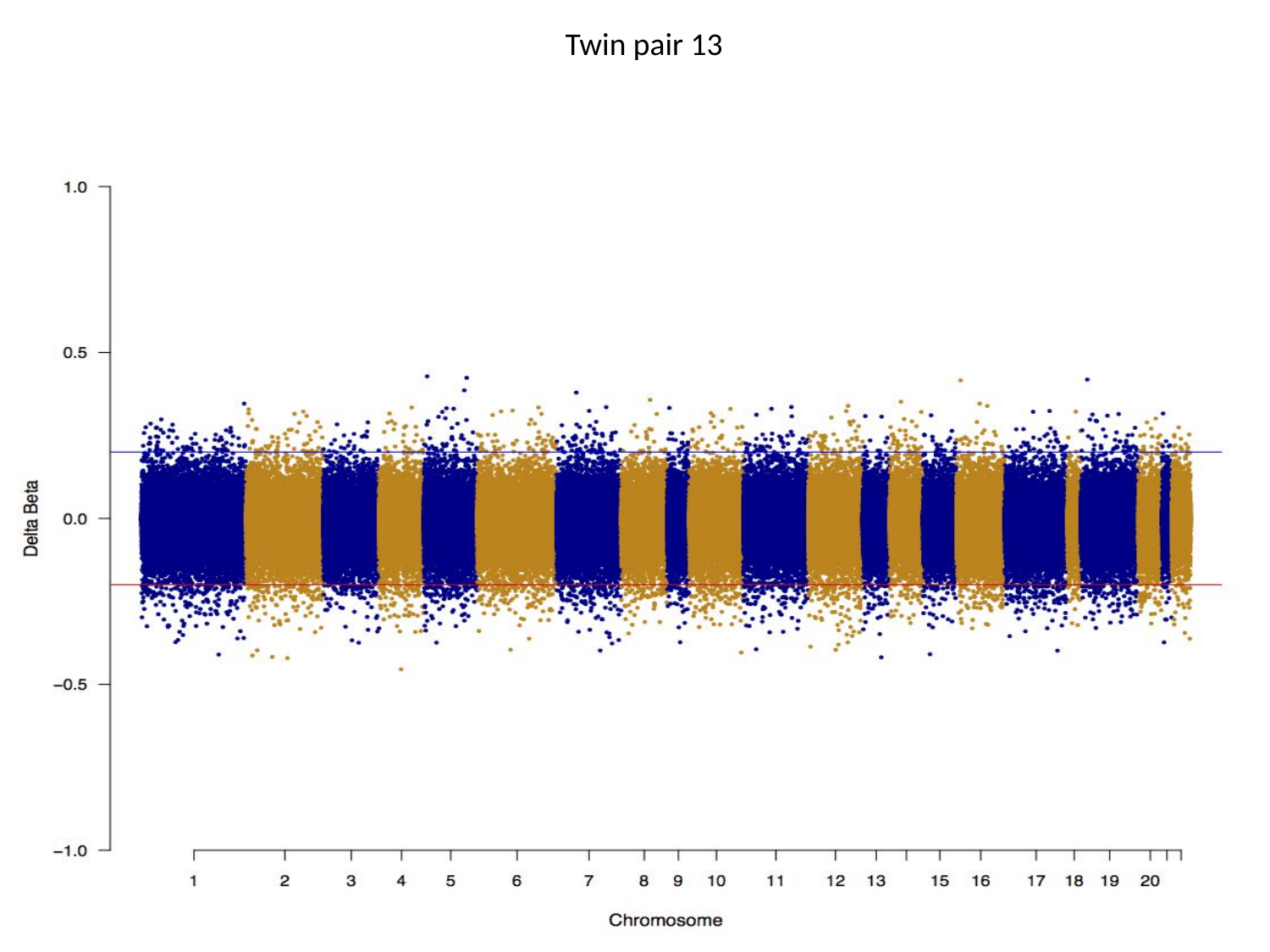

Twin pair 13

## Slide 13
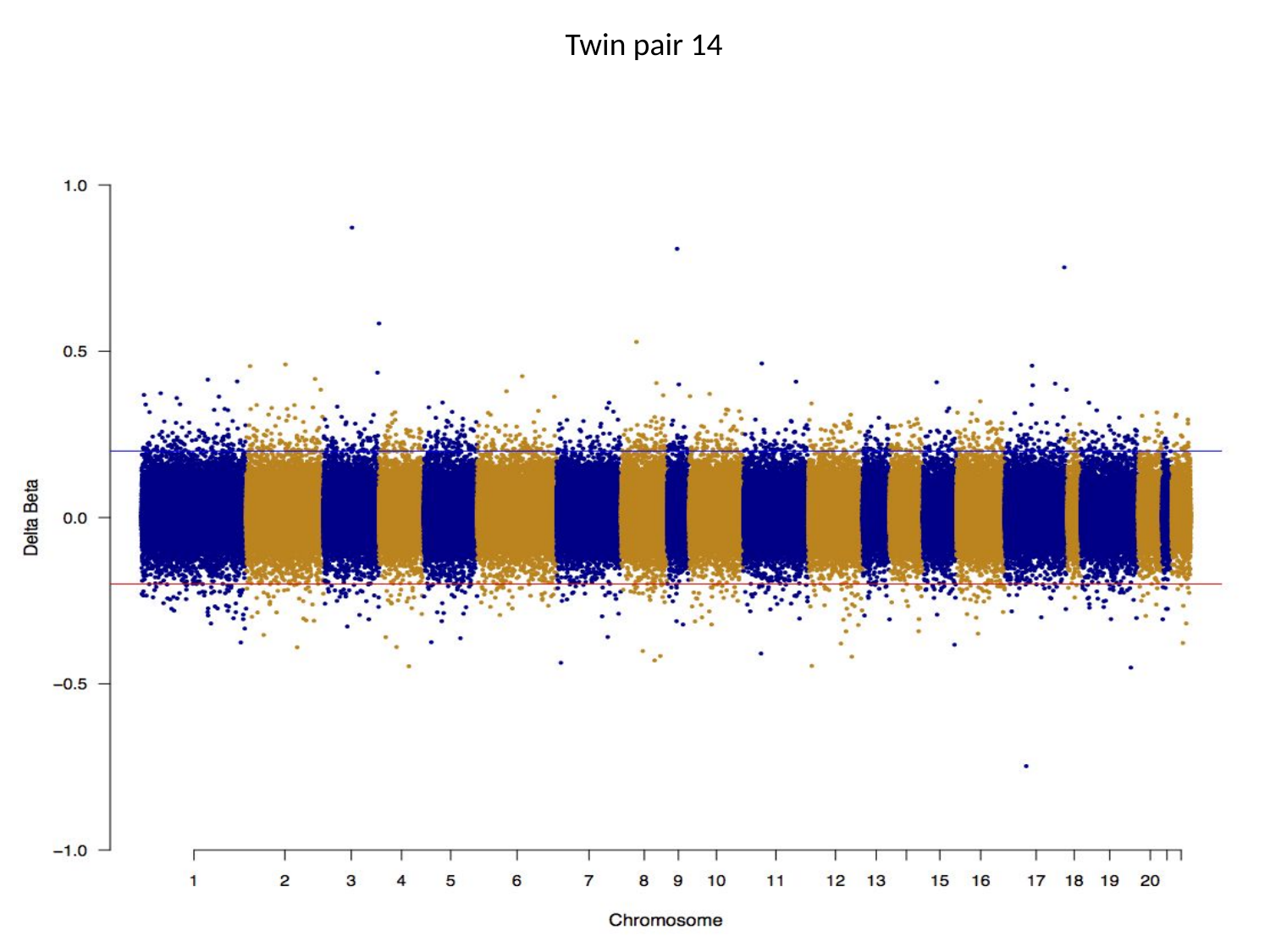

Twin pair 14

## Slide 14
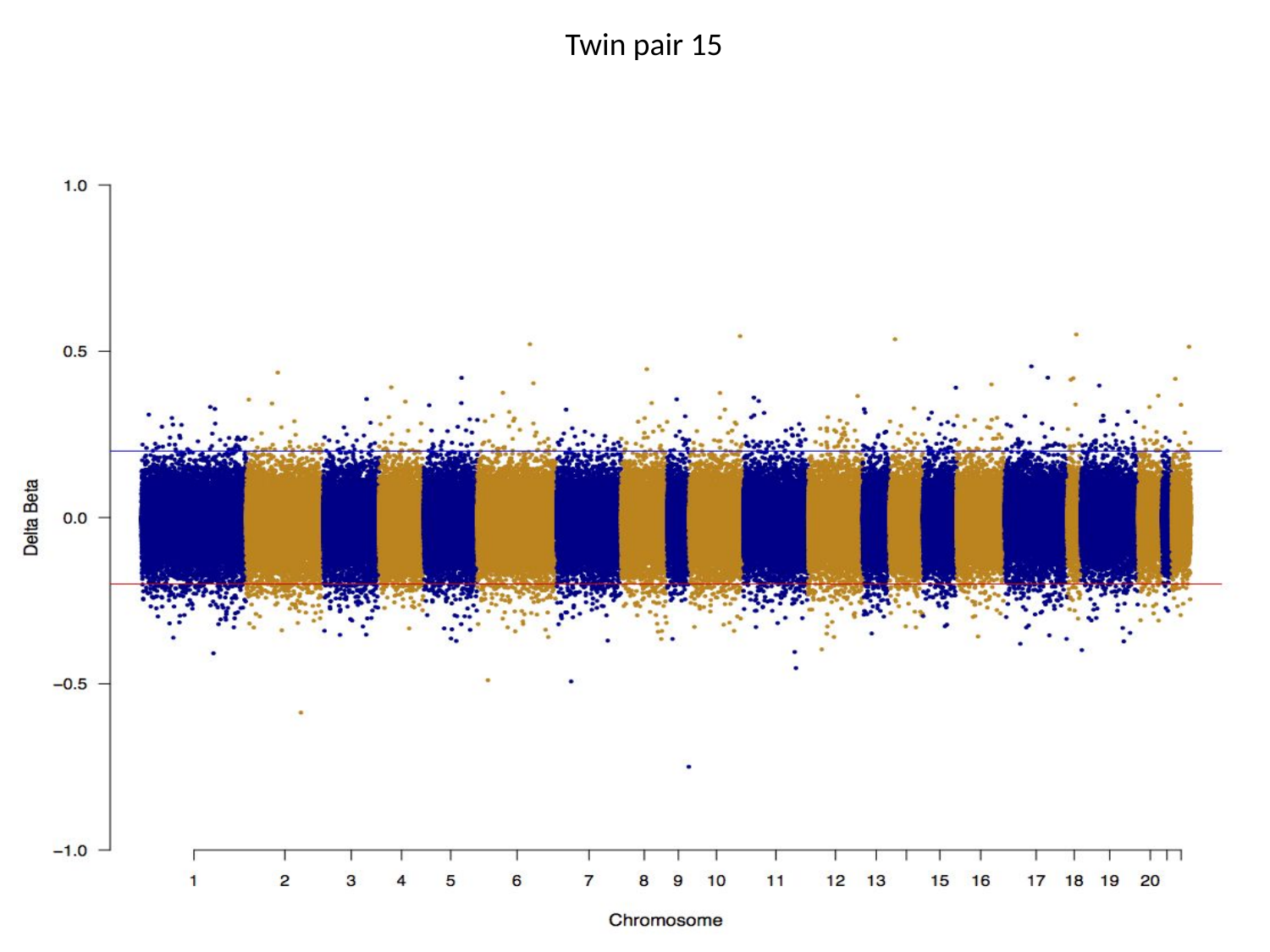

Twin pair 15

## Slide 15
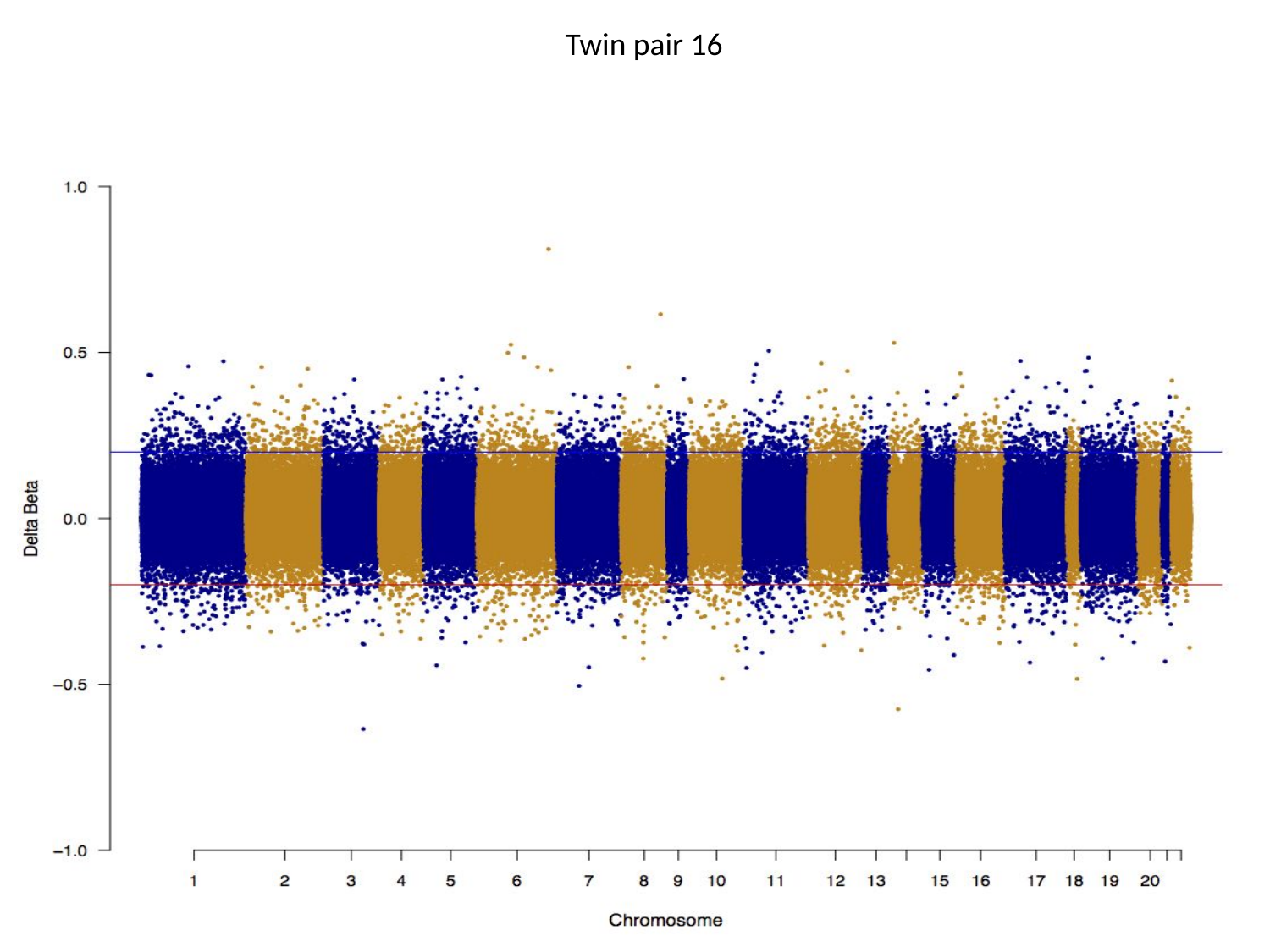

Twin pair 16
